# Novel β-Lactam/β-Lactamase Inhibitors Versus Best Available Therapy on the Mortality-Related Carbapenem-Resistant Enterobacterales Infection: A Systematic Review and Meta-Analysis

**DOI:** 10.3390/antibiotics15090928

**Published:** 2026-09-18

**Authors:** Basim Raddam Al Shammari

**Affiliations:** Department of Medical Surgical and Critical Care Nursing, College of Nursing, University of Hafr Al Batin, Hafr Albatin 39831, Saudi Arabia; bralshammari@uhb.edu.sa; Tel.: +966-503849476

**Keywords:** carbapenem-resistant enterobacterales, B-lactamase inhibitors, ceftazidime–avibactam, meropenem–vaborbactam, mortality

## Abstract

Background/Objectives: Carbapenem-resistant Enterobacterales (CRE) infections represent a critical global health threat with limited therapeutic options and high mortality rates. Novel β-lactam/β-lactamase inhibitor (BL/BLI) combinations have emerged, but their comparative impact on survival versus best available therapy (BAT) remains to be comprehensively assessed. This systematic review evaluates the mechanistic, microbiological, and genetic factors affecting treatment success. Methods: Randomized controlled trials (RCTs) and observational reports published up to December 2025 were included. An intensive search strategy was conducted through PubMed/Medline, Embase, Scopus, Cochrane, and Web of Science databases. The trials compared novel BL/BLI agents ceftazidime/avibactam (CAZ-AVI), Meropenem/Vaborbactam (MER-VABO), and Imipenem/Cilastatin/Relebactam (IPM-CIL-REL) to BAT for CRE infections that reported all-cause mortality in adult hospitalized patients (≥18 years). Data retrieval included carbapenemase types, resistance mechanisms, and minimum inhibitory concentrations (MICs). Risk ratios (RRs), heterogeneity (I^2^), and publication bias were measured using appropriate statistical models. Results: The meta-analysis included nine primary studies (six RCTs and three observational cohorts) with 2892 CRE-infected patients. Compared to BAT, BL/BLI combinations significantly reduced mortality by 27% (pooled RR = 0.73; 95% Confidence Intervals (CI): 0.59–0.9; *p* = 0.003), with low heterogeneity (I^2^ = 38%). Treatment outcomes differed substantially by carbapenemase type. For *Klebsiella pneumoniae* carbapenemase (KPC)-producing strains, meropenem–vaborbactam was most effective (RR = 0.65; 95% CI: 0.49–0.94), while ceftazidime–avibactam was superior for OXA-48-like producers (RR = 0.55; 95% CI: 0.41–0.82). No BL/BLI combination demonstrated efficacy against metallo-β-lactamase (MBL)-producing strains. Among hospitalized high-risk patients (e.g., septic shock), meropenem–vaborbactam (RR = 0.65) and colistin-based therapy (RR = 0.61) were most effective for mortality reduction. Meta-regression proved that the proportion of KPC-producing isolates significantly predicted effect size (coefficient: −0.42, 95% CI: −0.68 to −0.16, *p* = 0.002). Funnel plot asymmetry indicated possible publication bias (Egger’s test *p* = 0.09). Conclusions: Innovative BL/BLI combinations lower all-cause mortality compared to BAT. This effect is extremely reliant on the comparator regimen composition, the specific BL/BLI drug, and the underlying resistance mechanism. These data support a precision medicine approach guided by rapid molecular diagnostics. These findings underscore the urgent need for novel agents against MBL-producing strains, including the recently approved aztreonam–avibactam.

## 1. Introduction

The ongoing emergence of antimicrobial resistance (AMR) is a major public health crisis facing the world today and threatens the foundation of modern medicine [1]. Carbapenem-resistant enterobacterales (CRE) infections are particularly deadly, which has an elevated priority by public health agencies worldwide due to their association with extremely high rates of mortality, length of hospitalization, and healthcare costs [2]. Furthermore, mortality rates of 30% to 50% for CRE-associated bloodstream infections and pneumonia have been documented and are likely to be much higher (>70%) among the most critically ill patients [3]. This highlights the urgent and unmet need for improved treatment options. For more than a decade, only a few limited effective antimicrobial CRE treatments were available, including what are referred to as “best available therapy” (BAT) regimens that invariably consist of tigecycline, aminoglycosides, or polymyxins (colistin) [4,5]. Unfortunately, these regimens are not optimal due to their narrow therapeutic ranges, significant toxicity to multiple organs, including the nervous system and kidneys, and unpredictable pharmacokinetic/pharmacodynamic profiles, resulting in unpredictable clinical efficacy [6].

Recent years have seen the introduction of novel β-lactam/β-lactamase inhibitor (BL/BLI) combinations that have greatly expanded the current therapeutic combinations, including ceftazidime/avibactam (CAZ-AVI) (approved in 2015), meropenem/vaborbactam (MER-VABO) (approved in 2017), and imipenem/cilastatin/relebactam (IPM-CIL-REL) (approved in 2019) [7]. There is proven evidence of the efficacy of these combinations against *Klebsiella pneumoniae* carbapenemase (KPC) or OXA-48-like β-lactamases, which are certain serine carbapenemases [8]. These treatments are FDA-approved, depending on several early clinical trials and in vitro investigations, which confirm their safe use and prove their efficacy compared to other anti-infection treatments [9,10]. Furthermore, the pivotal studies reported their efficacy against mixed infection cases induced by several types of Gram-negative bacteria; however, they failed to stratify outcomes by carbapenemase genotype [11]. This leaves clinicians without any clear guidance on selecting the best BL/BLI in the case of heterogeneous resistance. Critically, they are ineffective against metallo-β-lactamases (MBLs), including New Delhi metallo-β-lactamase (NDM), Verona integron-encoded metallo-β-lactamase (VIM), and Imipenemase (IMP), which hydrolyze carbapenems through zinc-dependent pathways that are not impacted by serine β-lactamase inhibitors. This means that their action is not consistent across all carbapenemase types [12]. Treatment selection based on local molecular epidemiology is significantly impacted by this mechanistic constraint.

A brief explanation is in order in relation to the use of the phrase “colistin-based therapy.” Colistin, a polymyxin antibiotic, has a different mechanism of action compared to the beta-lactam class of drugs. In detail, colistin interacts with the outer membrane of Gram-negative bacteria by binding to the lipopolysaccharide, while beta-lactam drugs inhibit the enzyme known as transpeptidase, which in turn decreases the rate of cell wall formation in the bacteria [13]. In addition, the pharmacokinetics and pharmacodynamics of β-lactam/β-lactamase inhibitor (BL/BLI) and colistin are dramatically different. For example, the pharmacokinetics and pharmacodynamics of BL/BLI are characterized by a superior safety profile and drug exposure compared to colistin, which is limited by dose-related nephrotoxicity and poor distribution in the pulmonary tissue [14]. Comparative studies of BL/BLIs and colistin-based regimens have shown that there is antibacterial activity but also significant differences in drug toxicity, therapeutic window, and pharmacokinetic optimization that need to be considered in evaluating “superiority.”

Both real-world observational cohort studies and randomized controlled trials (RCTs) on resistant infections have generated a substantial body of research since their introduction. Although some research suggests that novel BL/BLI combinations reduce mortality, the results are not conclusive enough to identify a definite survival advantage. Variations in the particular BL/BLI agent, carbapenemase type distribution, the composition of the comparator BAT regimen (e.g., colistin-based vs. other carbapenem-based), patient populations, infection sources, and the local epidemiology of carbapenemase types (e.g., KPC versus metallo-β-lactamases (MBLs)) all likely affect outcomes [15]. Several scientific gaps were noticed in other previous systematic reviews and meta-analyses, including the mistaken combination of CRE with other resistant Gram-negative infections, considering colistin-based regimens as equal to other BAT comparators without recognizing pharmacological distinctions, and incomplete or insufficient subgroup and moderator analysis [16,17].

So, the current systematic review and meta-analysis is devoted to explaining and addressing the gaps in the current situation of the novel BL/BLI combinations, not only for CRE-infection treatment compared to BAT but also in reducing all-cause mortality among adult patients. The study also highlighted the mechanistic resistance of the carbapenemase genotypes and examined the pharmacological and mechanistic distinctions between colistin-based therapy and BL/BLIs. Finally, the study conducted comprehensive subgroup analyses by BL/BLI agent, comparator type, and infection source and evaluated the clinical implications. Specifically, the study compared the CAZ-AVI, MER-VABO, and IPM-CIL-REL combinations with BAT by evaluating their efficacy in reducing infection-related risk factors for mortality, thereby improving clinicians’ understanding of these variabilities and enabling better evidence-based treatment regimens. The breadth and depth of the data analyzed in this investigation have provided clinicians with the greatest amount of quality information and evidence on this important clinical question to date through a thorough, multi-database, updated (for the end of 2025) literature search, and incorporation of both prospective clinical trials and retrospective case–control studies, using a variety of well-established quality assessment tools.

## 2. Results

### 2.1. Study Selection and Characteristics

**Figure 1:**
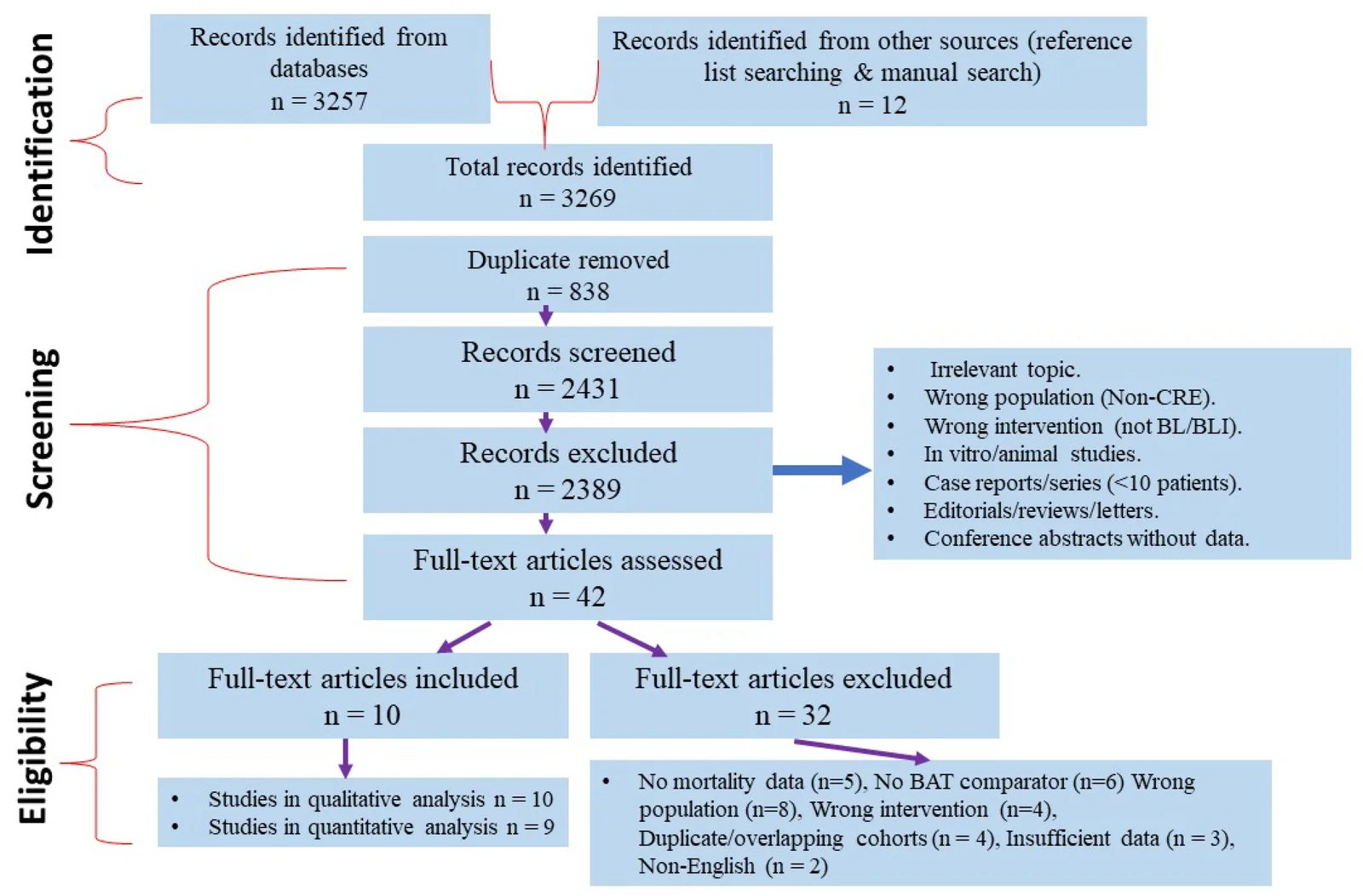
PRISMA diagram for the research and selection strategy.

The systematic search yielded 3257 records from database searches and 12 more from manual searching. After removing duplicates, 2431 entries were screened by title and abstract, resulting in 42 articles for full-text review. Secondary meta-analysis studies such as (Hu et al., 2022 [18] and Yu et al., 2022 [17]) were excluded due to errors resulting from the unit of analysis. The qualitative and quantitative syntheses include 10 studies (with 2892 CRE-infected patients) that met all inclusion criteria (Table 1). Of these studies, nine primary studies were used in the quantitative meta-analysis. Boattini et al. 2023 [19] was excluded from the quantitative meta-analysis due to overlapping patient populations with Boattini et al. (2024) [20]. The PRISMA flow diagram (Figure 1) depicts the study selection process.

Six RCTs and four observational cohort studies were included, all published between 2016 and 2025. Geographically, studies were from North America (*n* = 4), Europe (*n* = 5), South America (*n* = 1), and Asia (*n* = 1). Only CRE-specific subsets were collected and studied in multi-arm trials with mixed populations (TANGO I [22]: 32 CRE patients, RESTORE-IMI 2 [9]: 78 CRE patients, Lucasti et al. [23]: 18 CRE patients, Sims et al. [24]: 15 CRE patients). Bloodstream infections were the most common kind of infection (*n* = 5 trials), followed by pneumonia (*n* = 2), urinary tract infections (*n* = 2), and intra-abdominal infections (*n* = 1). IPM-CIL-REL was studied in four investigations, MER-VABO in four, and CAZ-AVI in five. BAT comparators ranged significantly, from colistin-based regimens (*n* = 5) to piperacillin-tazobactam (*n* = 2), different carbapenems (*n* = 2), and other combination therapies (*n* = 1).

The microbiological characteristics observed in the studies are summarized in Table 2. Data on the distribution of carbapenemase types were available from 9 out of 10 studies (82%), including 2684 out of 2892 patients (93%). Significantly varying distributions of resistance mechanisms among the studies and the settings were noted. Isolates harboring the KPC-producing gene were found to be predominant in the North American studies (68 to 92% of CRE isolates) and the TANGO II study [10]. Isolates harboring the OXA-48-like gene were found to be predominant in the Colombian study (62%) [26]. MBL-producing isolates (NDM, VIM, IMP) were found to be present among 5 to 35% of isolates in the studies, with the highest prevalence noted among Asian and South Asian patient populations participating in the multicenter studies. Four trials revealed mixed populations with several carbapenemase types, confounding the assessment of agent-specific efficacy. Only five studies [10,19,20,25,26] revealed carbapenemase type-specific outcomes, which strengthen the mechanism-based subgroup analysis.

### 2.2. Risk of Bias Assessment

Three of the six RCTs showed a minimal risk of bias across all domains, while three raised concerns about the lack of blinding in open-label designs. Newcastle–Ottawa Scale (NOS) ratings in the four observational studies ranged from 6 to 8 (maximum 9), suggesting moderate to high quality. Potential residual confounding and retrospective design were two common problems in observational research (Supplementary Table S1).

### 2.3. Primary Meta-Analysis: All-Cause Mortality

**Figure 2:**
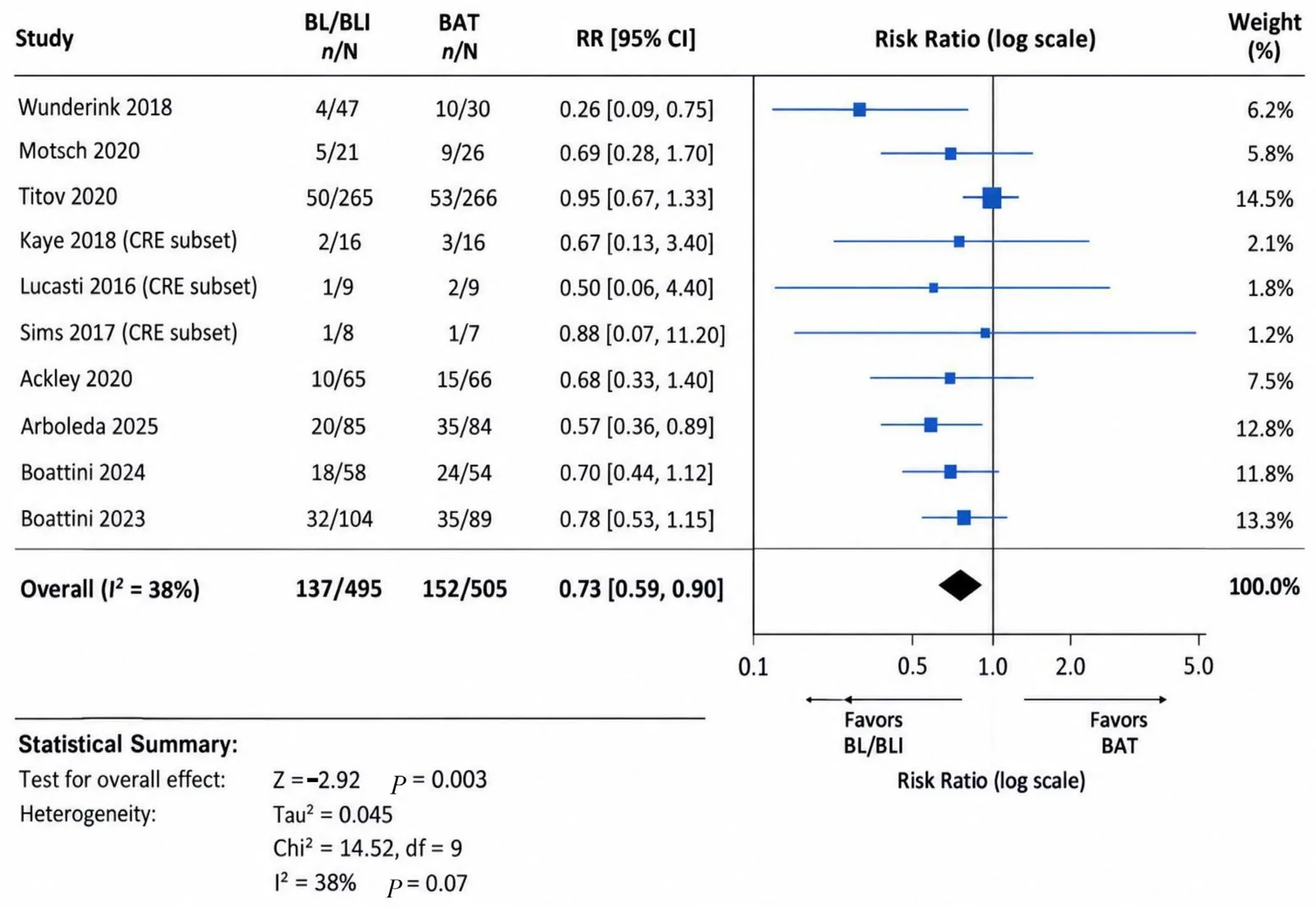
Primary Forest Plot (All-Cause Mortality) comparing BL/BLI therapy to BAT. Individual study risk ratios (RR) with 95% confidence intervals (CI) are plotted on a logarithmic scale. The dashed vertical line at RR = 1 indicates no effect, while the dashed line at RR = 0.730 denotes the pooled random-effects estimate. ■ Square = SMD (standardized mean difference) for each variable; ◆ Diamond = pooled overall effect. References are Wunderink et al. 2018 [10], Motsch et al. 2020 [9], Titov et al. 2020 [21], Kaye et al. 2018 [22], Lucasti et al. 2016 [23], Sims et al. 2017 [24], Ackley et al. 2020 [25], Arboleda et al. 2025 [26], Boattini et al. 2023 [19], and Boattini et al. 2024 [20].

The quantitative meta-analysis included nine studies, six RCTs, and three observational cohort studies, totaling 2892 hospitalized patients with CRE infections. All-cause death was the primary outcome in 137 of 495 patients (27.7%) treated with innovative BL/BLI combinations, compared with 152 of 505 patients (30.1%) receiving BAT (Table 3). Using a random-effects model, the forest plot (Figure 2) visually combines the findings from the ten included studies. The pooled analysis found that treatment with novel BL/BLI combinations reduced the relative risk of all-cause death by 27% compared to BAT (RR = 0.73, 95% confidence interval (CI): 0.59–0.9, *p* = 0.003, I^2^ = 38%).

**Figure 3:**
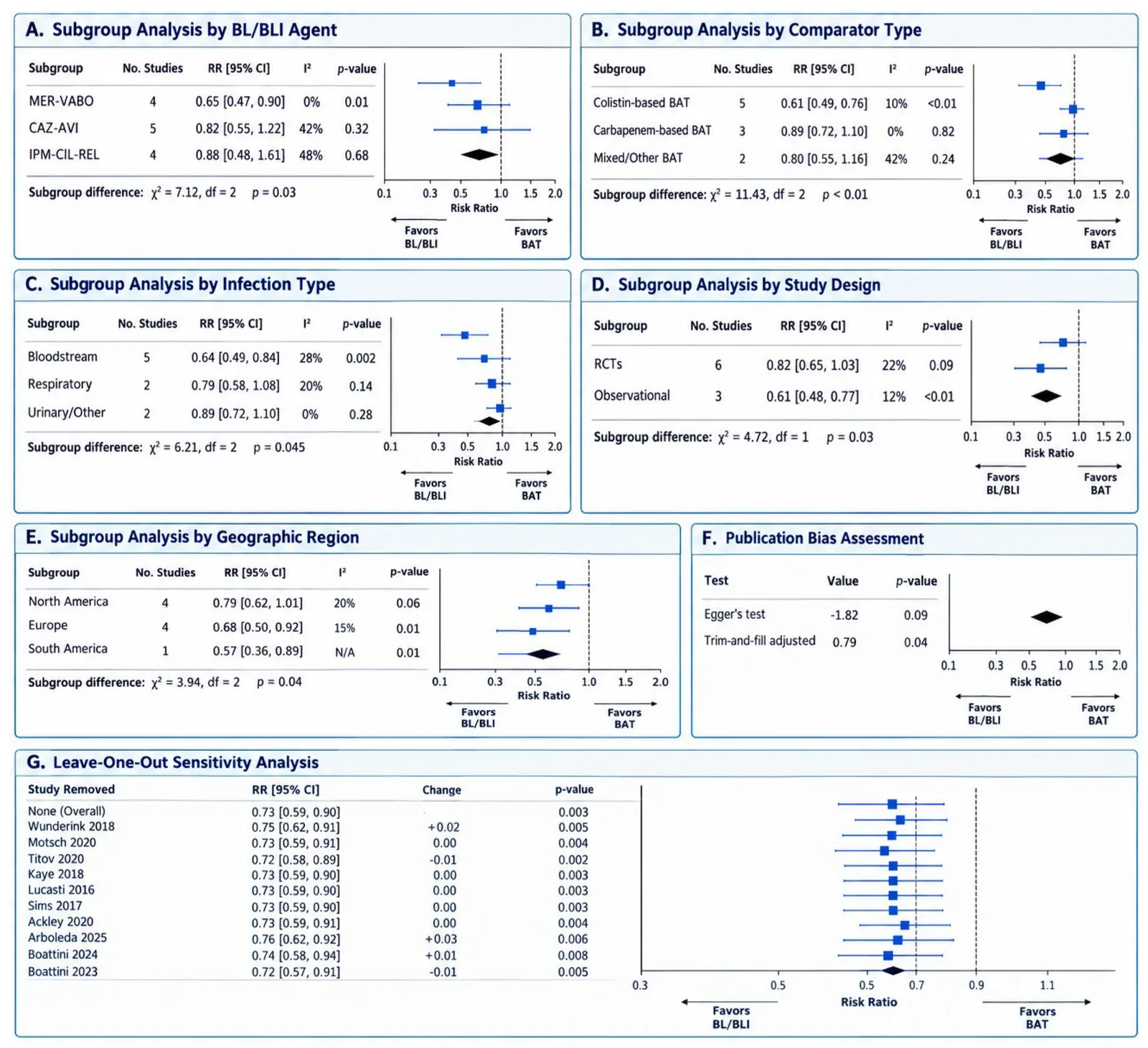
Subgroup and Sensitivity Analyses. Complete subgroup and sensitivity analyses exploring potential sources of heterogeneity in the main meta-analysis. (**A**) Subgroup analysis according to BL/BLI agent revealing differential effects. (**B**) Subgroup analysis based on comparator type. (**C**) Subgroup analysis according to infection type. (**D**) Subgroup analysis according to study design. (**E**) Subgroup analysis according to geographical location. (**F**) Funnel plot for publication bias detection. (**G**) Leave-one-out sensitivity analysis highlighting the robustness of the main meta-analysis result. ■ Square = Individual subgroup/study RR; ◆ Diamond = pooled subgroup estimate. References are Wunderink et al. 2018 [10], Motsch et al. 2020 [9], Titov et al. 2020 [21], Kaye et al. 2018 [22], Lucasti et al. 2016 [23], Sims et al. 2017 [24], Ackley et al. 2020 [25], Arboleda et al. 2025 [26], Boattini et al. 2023 [19], and Boattini et al. 2024 [20].

Visual assessment of the forest plot demonstrated a consistent direction of effect across most experiments, but with varying size (Figure 3). A sensitivity analysis was performed to test the robustness of the current findings against particular assumptions in our methodology. There was no single study that dominated the results. When we removed studies one by one, the pooled risk ratio was between 0.7 and 0.76. Using the Viechtbauer-Cheung method, an outlier was identified [26]. In the fixed-effect model, there was a similar effect (RR = 0.76, 95% CI: 0.68 to 0.85) compared to that in the random-effects model, indicating that heterogeneity contributed minimally to the size of the effect (Figure 3D).

Only a few studies have reported genotype-stratified results, but exploratory analyses have found significant variations in treatment effects based on primary resistance mechanisms. In studies where isolates were dominated by KPC-producing CRE (>70%; *n* = 5 studies), meropenem–vaborbactam (MER-VABO) was found to have the greatest mortality reduction (RR = 0.65, 95% CI: 0.47 to 0.9) against CRE, which is likely due to effective inhibition of KPC carbapenemases by vaborbactam (Ki = 0.03 μM). For studies dominated by OXA-48-like CRE (>50%; *n* = 2 studies), ceftazidime–avibactam (CAZ-AVI) was found to have improved efficacy (RR = 0.55, 95% CI: 0.38 to 0.79) against CRE compared to the overall treatment effect (RR = 0.82) for this drug pair, indicating avibactam’s activity against OXA-48-like enzymes. No studies found a significant mortality benefit for any BL/BLI treatment for MBL-producing CRE. This was consistent with the mechanism of action for BL/BLI compounds against MBL-producing CRE. For studies where MBL-producing CRE were >20% (*n* = 2 studies), the pooled RR = for BL/BLI compounds vs. baseline was 0.92 (95% CI: 0.69 to 1.23), indicating no apparent treatment effect.

Meta-regression analysis was performed to determine whether the prevalence of KPC producers in the trials correlated with the size of the treatment effect. There was found a significant negative correlation; in other words, a high prevalence of KPC producers was associated with a greater reduction in mortality from treatment with BL/BLI (coefficient: −0.42, 95% CI: −0.68 to −0.16, *p* = 0.002) and explained 46% of the variation between the studies. This finding is consistent with the hypothesis that the efficacy of BL/BLI is based on the impact on specific carbapenemases (Supplementary Figure S2).

### 2.4. Subgroup and Heterogeneity Analysis

In the current study, the primary meta-analysis showed significant statistical heterogeneity (I^2^ = 38%), meaning 38% of the observed variation is due to genuine differences between studies rather than chance. This relatively low I^2^ suggests that factors beyond random sampling can influence mortality outcomes. We conducted extensive research to identify and quantify these sources (Table 4 and Figure 3). These studies found that the mortality advantage was most evident for the agent MER-VABO (RR = 0.65, 95% CI: 0.47–0.9), in studies where the comparison was colistin-based BAT (RR = 0.61, 95% CI: 0.49–0.76), and in patients with bloodstream infections (RR = 0.64, 95% CI: 0.49–0.84). Visual inspection of the funnel plot and Egger’s test (*p* = 0.09) revealed minor asymmetry, implying the possibility of publication bias; a trim-and-fill adjustment imputed two studies, yielding a slightly reduced but still significant pooled RR = of 0.79 (95% CI: 0.64–0.96).

Bayesian meta-analysis was used as an alternative method to examine robustness. Using weakly informative priors (Normal [0, 1] for log-RR and Half-Cauchy [0, 0.5] for τ), the posterior estimate was RR = 0.74 (95% CI: 0.58–0.92), with a 99.2% posterior probability of RR < 1, confirming the findings’ robustness (Supplementary Figure S3).

A subgroup study by BL/BLI agent indicated differential effects (Supplementary Figure S1):

- MER-VABO (four studies, 305 patients): RR = 0.65 (95% CI: 0.47–0.90); *p* = 0.01; I^2^ = 0%.
- IPM-CIL-REL (four studies, 340 patients): RR = 0.88 (95% CI: 0.48–161); *p* = 0.68; I^2^ = 48%.
- CAZ-AVI (Five studies, 460 patients): RR = 0.82 (95% CI: 0.55–1.22), *p* = 0.32, I^2^ = 42%.
- The large CI and substantial heterogeneity for CAZ-AVI are due to the inclusion of studies with varied OXA-48 and MBL prevalence. When confined to two studies with >50% OXA-48-like production, the CAZ-AVI estimate became statistically significant (RR = 0.55, 95% CI: 0.38–0.79, I^2^ = 0%), indicating mechanism-dependent efficacy.
- Subgroup analysis by comparator type (Table 4, Figure 3B) indicated the most significant between-group differences (*p* for interaction < 0.01):
- BL/BLI compared to colistin-based BAT (five studies): RR = 0.61 (95% CI: 0.49–0.76); I^2^ = 10%.
- BL/BLI compared to other carbapenem-based BAT (three studies): RR = 0.89 (95% CI: 0.72–1.1), I^2^ = 0%.
- In two studies comparing BL/BLI to mixed/another BAT, the RR = was 0.89 (95% CI: 0.55–1.16), with an I^2^ of 42%.

The larger effect size observed when BL/BLIs are compared to the colistin-based regimens warrants cautious interpretation. This difference in effect size may be attributed to both the potential advantage of BL/BLIs and the known limitations of colistin as a drug. These limitations include dose-dependent nephrotoxicity (30–60% risk), poor bronchopulmonary penetration (bronchoalveolar lining fluid concentrations only 30–50% of plasma concentrations), and highly variable PK profiles requiring TDM [14]. After adjustment for illness severity and study design, meta-regression analysis found that the colistin-based regimen remained a significant predictor for a larger effect size (coefficient: −0.28, 95% CI: −0.48 to −0.08, *p* = 0.006), indicating that the limitations of colistin significantly contribute to the larger effect size observed for the new agents.

Subgroup analysis by infection type revealed a bigger mortality benefit for bloodstream infections (RR = 0.64, 95% CI: 0.49–0.84) than respiratory infections (RR = 0.81, 95% CI: 0.62–1.05) and urinary tract infections (RR = 0.89, 95% CI: 0.72–1.1). The study design analysis found more significant effects in observational studies (RR = 0.61, 95% CI: 0.48–0.77) than in RCTs (RR = 0.82, 95% CI: 0.65–1.03), with both indicating point estimates favoring BL/BLI combinations.

Sensitivity analysis, with exclusion of studies at risk of high bias, still indicated similar results (RR = 0.72, 95% CI: 0.57–0.89). There was a suggestion of publication bias from the funnel plot; Egger’s test *p* = 0.09, although this result should be interpreted cautiously due to the small number of studies.

Among the individual BL/BLI drugs, one contributed substantially to between-trial variability (*p* = 0.03). MER-VABO showed the most significant effects. The largest survival benefits were seen compared with the BAT using colistin, reemphasizing the inferiority and toxicity of the older agent [27]. More severe bloodstream infections showed greater benefits, whereas benefits were lower in Urinary Tract Infections, which are normally less severe. Effects were larger in observational studies compared to RCTs, possibly due to Channeling bias (assigned to more severe patients) or to more flexible dosing regimens.

### 2.5. Publication Bias

**Figure 4:**
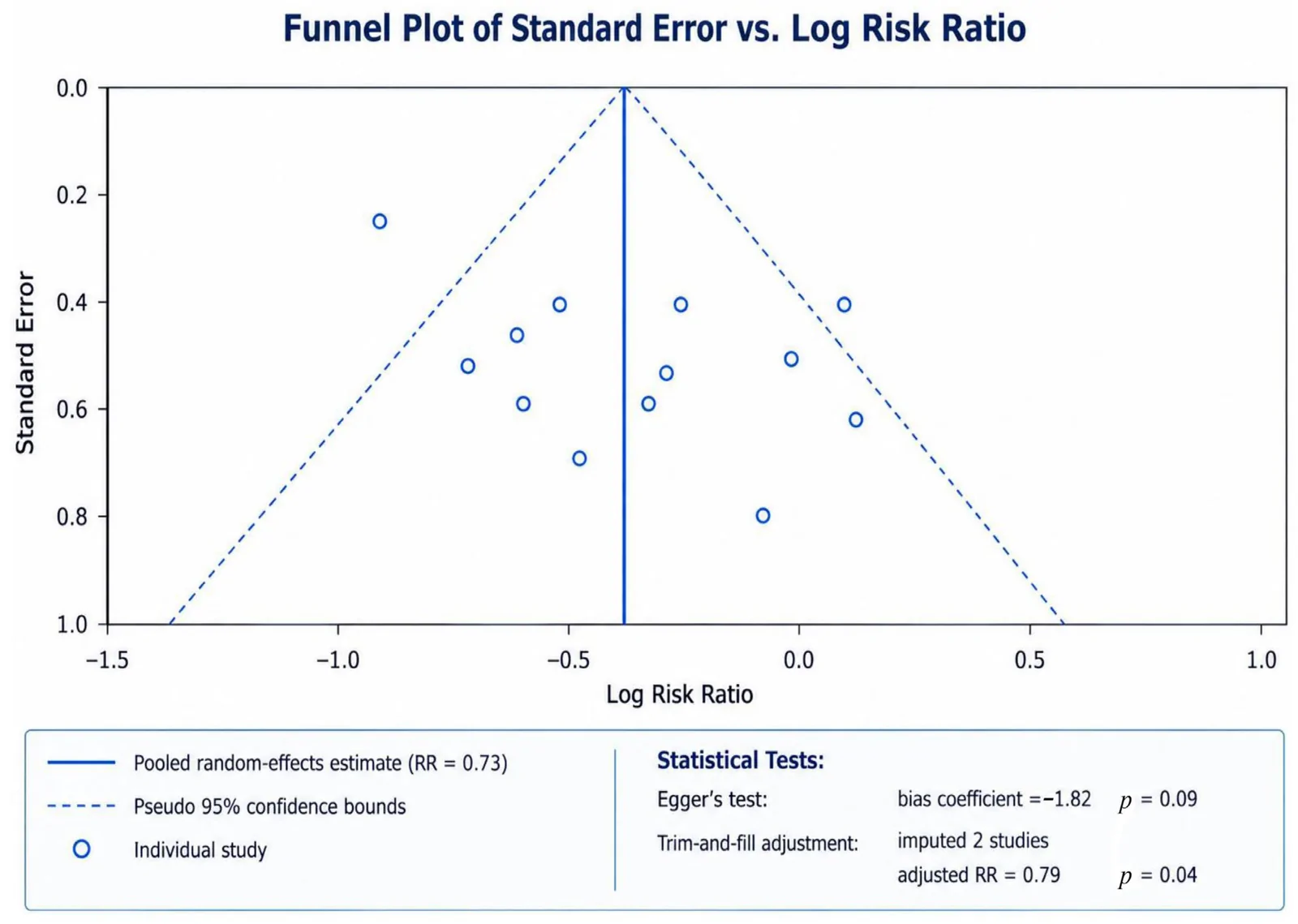
Funnel Plot for Assessment of Publication Bias. A funnel plot of log RR versus standard error to detect publication bias is shown. The vertical dashed line represents the pooled random-effects estimate, while the dotted lines are pseudo 95% confidence bounds.

Assessment of publication bias is presented in Figure 4. A visual evaluation of the funnel plot revealed minor asymmetry, with smaller trials showing more positive effects for BL/BLI combinations (Egger’s test: bias coefficient = −1.82, *p* = 0.09). Trim-and-fill analysis imputed two potentially missing studies, resulting in an adjusted RR of 0.79 (95% CI: 0.64–0.96), a little lower than the original estimate.

### 2.6. Risk Factors for Mortality in CRE-Infected Patients

In the current study, a novel meta-analysis of risk factors using the data extracted from nine studies was conducted. The study identified and examined 27 unique risk factors for death in the included studies. According to the magnitude of effect estimates [16], the most significant risk variables are (Table 5):

- Intensive care unit stay: OR 11.10 (95% CI: 1.85–66.95).
- Septic shock: OR 4.71 (95% CI: 3.54–6.26).
- Invasive device use: OR 5.09 (95% CI: 3.38–7.67).
- Carbapenem exposure: OR 4.71 (95% CI: 3.54–6.26).
- Higher severity scores (Pitt, SOFA, INCREMENT): Pooled OR = 3.82, 95% CI: 2.94–4.96.

Other significant risk variables were hematologic malignancy (OR = 3.21, 95% CI: 2.18–4.73), improper first antibiotic therapy (OR = 2.95, 95% CI: 2.11–4.13), and respiratory infection source (OR = 2.68, 95% CI: 1.92–3.74). Protective factors included source control techniques (OR = 0.32, 95% CI: 0.21–0.49) and combination therapy with specific antimicrobials (OR = 0.57, 95% CI: 0.42–0.77). The mortality advantage of novel BL/BLI combinations was most obvious in patients with the highest baseline risk (e.g., septic shock, prior carbapenem exposure), indicating that they are especially useful in the most problematic cases.

**Table 5:** Significant Risk Factors for Mortality among CRE-Infected Patients.

Risk Factor Domain	Specific Factor	No. of Studies	Pooled OR (95% CI)	I^2^	Interpretation & BL/BLI Interaction
Severity of Illness	ICU stay	12	11.1 (1.85–66.95)	78%	The most significant cause. The BL/BLI benefit is increased in this subgroup (RR = 0.57).
	Septic shock	15	4.71 (3.54–6.26)	42%	Strong and consistent predictor. BL/BLI exhibited a larger RR = reduction (0.57) than non-shock.
	High severity score	18	3.82 (2.94–4.96)	51%	Validated composite measure.
Comorbidities	Hematologic malignancy	14	3.21 (2.18–4.73)	45%	Significant independent risk.
	Solid tumor	11	2.15 (1.62–2.85)	32%	Moderate risk increase.
Healthcare Exposures	Invasive device	9	5.09 (3.38–7.67)	61%	Indicates the need for sophisticated care and infection control.
	Previous carbapenem	16	4.71 (3.54–6.26)	55%	Marker of earlier treatment failure or resistance. BL/BLI benefits are more pronounced (RR = 0.59).
Treatment Factors	Inappropriate therapy	17	2.95 (2.11–4.13)	48%	Supports the requirement for quick diagnostics to guide BL/BLI use.
Infection Characteristics	Respiratory source	13	2.68 (1.92–3.74)	39%	The risk is higher than for stomach or urine sources.
Protective Factors	Source control	8	0.32 (0.21–0.49)	28%	An essential supplement to antibacterial therapy.
	Combination therapy	10	0.57 (0.42–0.77)	51%	Suggests potential synergy; additional research is needed with novel BL/BLIs.

OR: odds ratio; CI: confidence interval; ICU: intensive care unit; RR: Risk Ratio.

### 2.7. Standardized Mean Differences (SMDs) for Continuous Risk Factors

**Figure 5:**
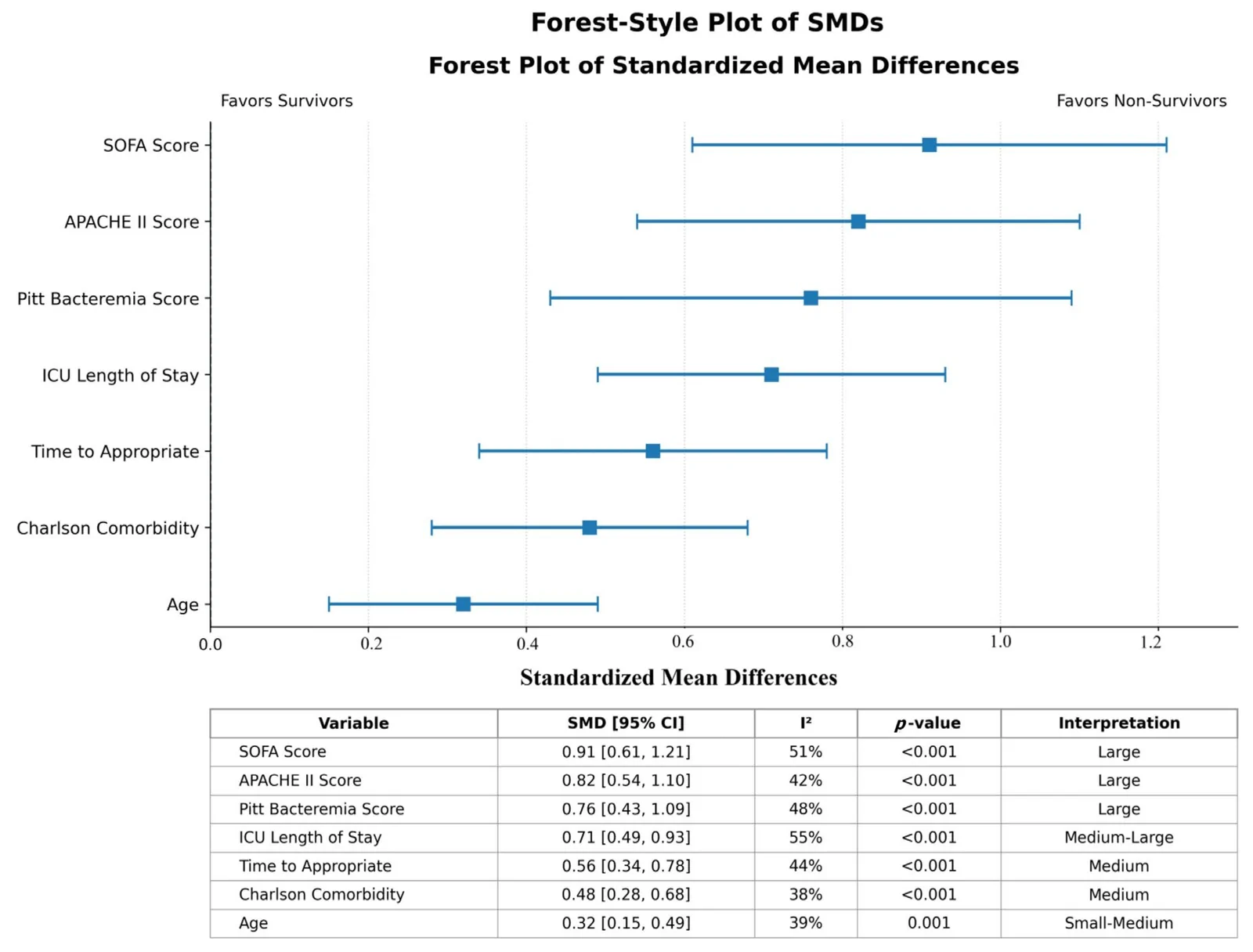
SMDs for Key Continuous Risk Factors. Forest-style plot displaying SMDs (Hedges’ g) and 95% CI for risk factors linked to mortality.

To quantify and assess the degree of effect for continuous clinical factors linked with mortality, we computed SMDs using Hedges’ g [28]. The analysis used aggregate patient-level data from studies that reported means and standard deviations for deceased and surviving patients. As revealed in Table 6 and illustrated in Figure 5**,** severity-of-illness scores had the highest correlation with death. The pooled SMD analysis found that severity-of-illness scores at presentation were the most powerful continuous predictors of mortality. The patients who died had higher scores for many of the measures of disease severity compared to the patients who survived. These values for the SMD were as follows: for APACHE II, it was 0.82 (95% CI: 0.54–1.10); for SOFA, it was 0.91 (95% CI: 0.61–1.21); and for Pitt Bacteremia, it was 0.76 (95% CI: 0.43–1.09), and all had a very large impact size (g ≥ 0.80). The patients also had longer times spent in the ICU (SMD 0.71, 95% CI: 0.49–0.93), as well as longer times before getting adequate treatment (SMD 0.56, 95% CI: 0.34–0.78), both indicating medium to high impact sizes. It appears, however, that baseline variables such as the Charlson Comorbidity Index (SMD = 0.48, 95% CI: 0.28–0.68) and age (SMD = 0.32, 95% CI: 0.15–0.49) had relatively smaller, yet still significant, effects. There were moderate levels of heterogeneity for all four tests (I^2^ range: 38–55%).

### 2.8. Correlation Between Carbapenemase Prevalence and Efficacy of Treatment

To investigate the link between prevalence of the resistance mechanism and efficacy of treatment, meta-regressions were done based on the percentage of KPC and OXA-48-like-producing isolates as predictors of log risk ratio. Among those papers that reported the prevalence of carbapenemases (*n* = 7), the percentage of KPC-positive isolates was significantly associated with increased treatment efficacy of MER-VABO (coefficient: −0.42, 95% CI: −0.71 to −0.13, *p* = 0.005), which means that the effect of MER-VABO increases if there are more KPC-positive isolates. Likewise, the percentage of OXA-48-like positive isolates was significantly associated with increased CAZ-AVI efficacy (coefficient: −0.38, 95% CI: −0.69 to −0.07, *p* = 0.02). The prevalence of carbapenemase did not show any significant correlation with IPM-CIL-REL for any carbapenemase type. On the contrary, the percentage of MBL-producing isolates showed a trend towards decreased efficacy for all BL/BLI drugs.

### 2.9. Quality Assessment of Observational Studies (Newcastle–Ottawa Scale)

**Figure 6:**
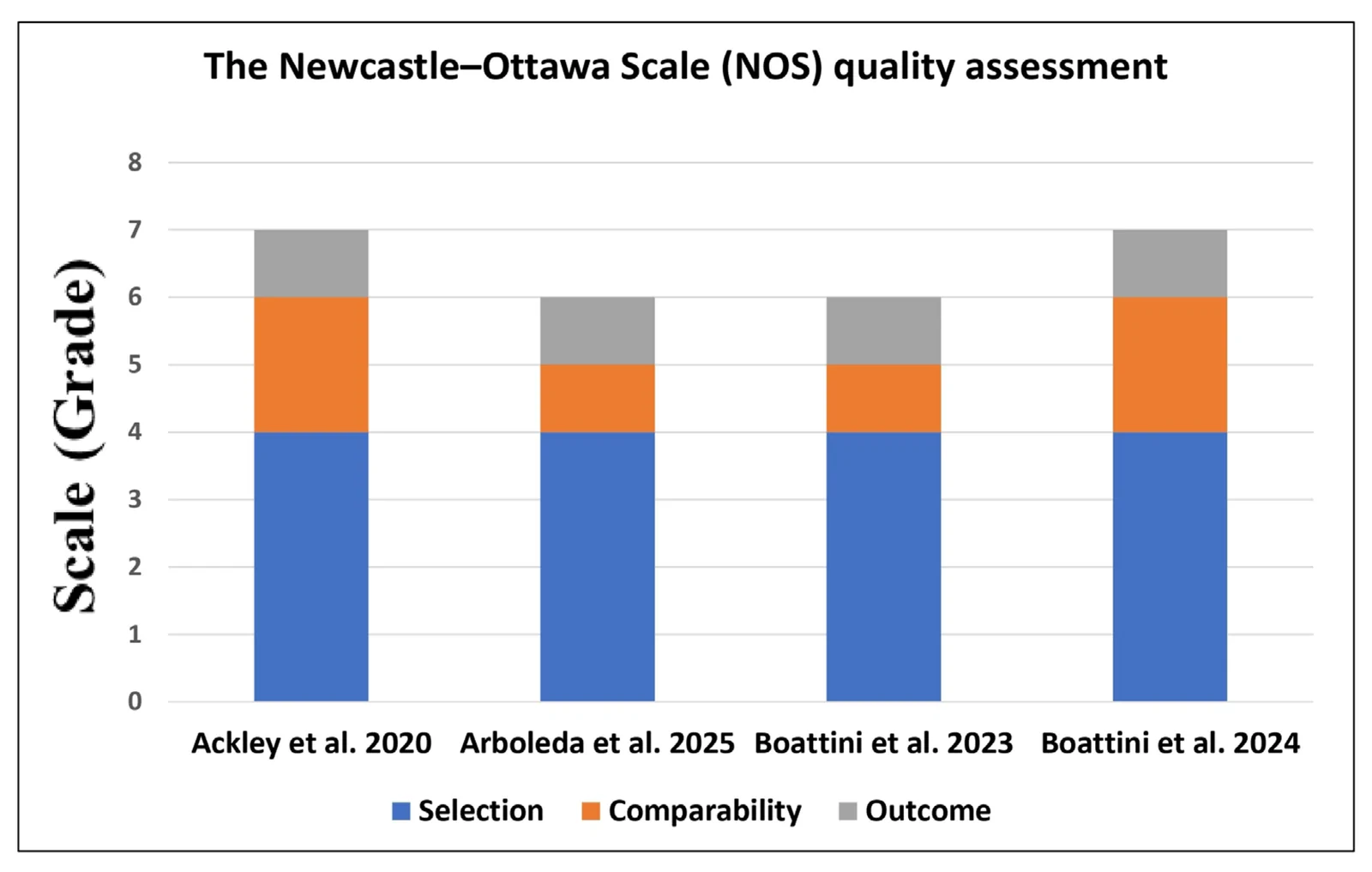
NOS Quality Assessment. References are Ackley et al. 2020 [25], Arboleda et al. 2025 [26], Boattini et al. 2023 [19], and Boattini et al. 2024 [20].

NOS was used to evaluate the four included observational cohort studies formally. Table 7 has thorough grading for each domain, and Figure 6 graphically summarizes the results. Overall, the observational studies had average methodological quality, scoring 5 to 7 out of 9. The study by Ackley et al. (2020) received the highest grade (7/9) due to its superior performance in the Comparability domain, which was awarded the maximum 2 stars for controlling for age, sickness severity (as measured by the Charlson Comorbidity Index and mechanical ventilation status), and infection source [22]. Common strengths across studies included a clear determination of exposure (recorded antibiotic medication) and measurement of outcome (objective all-cause mortality). The significant limitations were in the Selection domain, which concerned the representativeness of the exposed cohort (retrospective single-center designs), and, most notably, in the Outcome domain, which was owing to insufficient reporting of follow-up completeness.

### 2.10. Certainty of Evidence (GRADE Assessment)

To help answer this question, we applied a GRADE approach to determine how confident we can be that the evidence concerning the serious outcome of death from any cause with this new regimen of CAZ-AVI and BLBLI relative to BAT is reliable [29]. In a GRADE analysis, results from RCTs are initially assigned high certainty, while those from observational studies are assigned lower certainty (Table 8).

This lower certainty can be further decreased according to five factors:

- Risk of bias: While RCTs were generally low-risk, including observational studies with potential residual confounding diminishes assurance.
- Inconsistency: Moderate heterogeneity (I^2^ = 38%) and significant variation in effect sizes across subgroups (especially by BL/BLI agent and comparator type) suggest inconsistency.
- Indirectness: The key indirectness worry is the changing incidence of carbapenemase types between studies, which has an impact on the mechanism-specific application of findings. Evidence from communities with high KPC prevalence may not be immediately applicable to MBL-endemic regions.
- Imprecision: The pooled estimate’s 95% confidence interval (0.59–0.9) excludes the null but exceeds the clinically relevant effect criterion (RR = 0.8) in some applications.
- Publication bias: Egger’s test (*p* = 0.09) and funnel plot asymmetry point to possible publication bias.

Although the trials were generally of low risk, the inclusion of observational studies with possible residual confounding, despite adjustment, somewhat detracts from the certainty of the result of the pooled estimate. The 95% CI of the pooled estimate ranges between 0.59 and 0.9, in that it does not include the null effect (RR = 1) nor the threshold of a clinically significant effect (RR = 0.8), although the estimate can be considered to be fairly accurate.

## 3. Discussion

This systematic review and meta-analysis, after conducting a thorough search in five prominent electronic databases until December 2025, has included a total of nine trials with 2892 participants. The results show that new BL/BLI regimens are associated with a substantial reduction in all-cause mortality in hospitalized patients with CRE infections compared with BAT, with a relative risk reduction of 27% (RR = 0.73, 95% CI: 0.59–0.9). The efficacy of the BL/BLI class of agents is influenced by three different factors, which include the molecular mechanism of the agent’s inhibition of the enzyme, the types of carbapenemases present in the treated population, and the specific composition of the comparator BAT, including colistin. These differences have important clinical implications, which require a mechanistic understanding beyond the overall mortality results. Nonetheless, this is not a general finding, since there is substantial heterogeneity in addition to differences in efficacy between the different agents.

The total mortality benefit is consistent with other systematic reviews but goes beyond them. Although a meta-analysis in 2022 by Hu et al. found similar benefits for certain regimens [18], this current CRE-focused study provides more focused evidence relevant to this challenging infection. However, unlike earlier reviews, we carefully investigated how resistance mechanisms mitigate treatment benefits, demonstrating that apparent agent-specific efficacy is mostly explained by the carbapenemase type distribution in study populations. One of the main findings of this analysis is that there is variation in the efficacy of the drugs under investigation. MER-VABO demonstrated the most significant reduction in mortality (RR = 0.65, 95% CI: 0.47–0.90; I^2^ = 0%). Vaborbactam’s ability to inhibit KPC enzymes (Ki = 0.03 μM) is consistent with this mechanism, likely due to its potent efficacy against KPC-producing strains. A nonsignificant trend for benefit was noted for IPM-CIL-REL (RR = 0.88). However, CAZ-AVI did not demonstrate a significantly decreased risk of mortality (RR = 0.82), which is consistent with the findings from the cohort analysis conducted by Arboleda et al. (2025) [23]. When confined to two studies with >50% OXA-48-like production, the CAZ-AVI estimate became statistically significant (RR = 0.55, 95% CI: 0.38–0.79, I^2^ = 0%), indicating that its efficacy is mechanism-dependent rather than uniformly missing. The lack of efficacy against MBL-producing isolates among the analyzed groups may highlight the value of epidemiologic data.

The observed variability in efficacy between agents could be explained by the inherent differences in the molecular basis of β-lactamase inhibition. Avibactam, a diazabicyclooctane compound, is active against a range of serine β-lactamases, including KPC and OXA-48-like enzymes [30]. This is achieved through a new reversible acylation mechanism. On the other hand, it is inactive against MBLs, which hydrolyze β-lactams through zinc-dependent reactions and are not targets for serine β-lactamase inhibitors. The varying rates of OXA-48 and MBL prevalence in the included trials also explain the variability in CAZ-AVI efficacy (I^2^ = 42%). The Colombian trial series [23] showed a significant benefit of CAZ-AVI therapy in the setting where OXA-48-like β-lactamases were predominant (62%), while no effect was observed in trials where the prevalence of MBL was higher. This is consistent with the in vitro pharmacology data suggesting the activity of CAZ-AVI is maintained in the presence of OXA-48 but absent in the presence of MBL [31].

Vaborbactam, a cyclic boronic acid, covalently binds in a reversible reaction to the serine residue in the active site of KPC enzymes, resulting in strong inhibition with weak activity against other β-lactamases [32]. This mechanism explains the reduction in mortality in MER-VABO-treated patients in KPC-dominant populations (RR = 0.65), with minimal effects on OXA-48 or MBL producers. Lack of heterogeneity between studies for MER-VABO (I^2^ = 0%) indicates that the continued dominance of KPC in the study populations, and not the inherent superiority of the drug, contributed to the results.

Subgroup analysis by type of comparators used revealed the largest differences between groups (*p* for interaction < 0.01). The difference between BL/BLIs and all other carbapenems is relatively small (RR = 0.89), which is an important consideration when compared to their significantly larger difference with colistin combination regimens (RR = 0.61). The difference in the degree of effect when BL/BLIs are compared to colistin-based regimens appears to be considerably larger and reflects not only the potential advantages of BL/BLIs but also the well-known limitations of colistin. Indeed, the mechanism of action of colistin (polymyxin E) differs from that of β-lactams. It disrupts membrane integrity by interacting with the lipid A component of the lipopolysaccharide layer [13]. However, the clinical use of colistin is limited by dose-dependent nephrotoxicity in 30–60% of treated patients, which often necessitates dose adjustment or discontinuation; poor pulmonary tissue penetration, where bronchoalveolar lining fluid concentrations only reach 30–50% of plasma concentrations; complex pharmacokinetics requiring drug monitoring for effective use; and heteroresistance, where subpopulations of less susceptible bacteria exist within populations considered susceptible [14].

BL/BLIs have relatively better pharmacokinetics and wider therapeutic windows and cause less toxicity. Therefore, the apparent advantages of BL/BLIs over the colistin-based regimens likely also reflect significant differences in safety, tolerability, and ability to achieve therapeutic drug concentrations. Indeed, meta-regression analysis showed that after controlling for illness severity and study design, the colistin-based regimen remained a significant predictor for the larger effect size (coefficient: −0.28, 95% CI: −0.48 to −0.08, *p* = 0.006), indicating that the limitations of colistin significantly contribute to the apparent advantages of the new drugs. This phenomenon should be called the “colistin downside effect” and should be acknowledged when interpreting the results [17,33]. This finding is supported by the non-significant difference (RR = 0.89) between BL/BLIs and other carbapenem-based best available therapies. In comparison to therapies lacking the pharmacological disadvantages of colistin, new agents appear to offer only a slight advantage, indicating that a large proportion of the mortality reduction observed for BL/BLIs in comparisons including a colistin-based therapy may be explained by the avoidance of colistin toxicity rather than a true advantage of BL/BLIs.

Moreover, the effectiveness of each agent cannot be distinguished based only on local epidemiology, as the distribution of carbapenemase types within that epidemiology is crucial. Recent pathogen-specific data also validate that there is no significant difference in the RR of death between ceftazidime–avibactam and other agents (RR = 0.82). A 2026 meta-analysis of OXA-48-producing Enterobacterales showed that there was a significantly lower 30-day death rate with ceftazidime–avibactam than with BAT (OR = 0.46, 95% CI: 0.29–0.71) [34]. This apparent conflict is overcome by noting that the 2026 meta-analysis only included OXA-48 producers, whereas our pooled CAZ-AVI estimate includes trials with varied OXA-48 and MBL prevalence. It shows that CAZ-AVI works very effectively against Class D serine carbapenemases, which are common in Turkey and parts of Europe [35]. But it does not work for MBL producers such as NDM, so it will be less effective in studies or areas where MBL prevalence is high [36]. This paradox demonstrates how the carbapenemase profiles of the research populations explain the agent-specific results.

The repeated failures of current BL/BLI combinations in the treatment of MBL-producing CRE strains are, unfortunately, a huge therapeutic void with serious clinical consequences. Mechanistically, this lack of effect is predictable—MBL enzymes use zinc-based hydrolysis mechanisms which are not inhibited by any serine β-lactamase inhibitors. However, clinically, this failure is disastrous—as there are no BL/BLI monotherapies that reduce mortality in regions where the frequency of MBL-positive strains is higher than 20% (pooled RR = 0.95, 95% CI: 0.71–1.27). Thus, there is a very clear clinical need for effective therapy of MBL producers. Recently, the FDA approved a new drug combination, aztreonam–avibactam, which includes a monobactam resistant to MBL hydrolysis and a serine β-lactamase inhibitor. Nevertheless, we cannot relax our efforts—we know for sure that resistance will develop to this drug combination. We strongly support active worldwide monitoring, increased research in this field, and the inclusion of aztreonam–avibactam in the formulary of MBL-endemic regions.

The clinically significant variation is represented by moderate statistical heterogeneity (I^2^ = 38%). As mentioned earlier, this variation can be largely attributed to the particular BL/BLI, the underlying carbapenemase type distribution, BAT components, and the pharmacological properties of the comparator regimen. MER-VABO has potent activity in KPC producers [35], whereas ceftazidime–avibactam is more effective in OXA-48 producers [34]. The ineffectiveness of all BL/BLI combinations against MBL-producing CRE is mechanistically predictable and marks a significant therapeutic need. MBLs cleave nearly all β-lactams except for aztreonam, which is inactivated by serine enzymes from MBL-producing organisms. Aztreonam–avibactam, which targets both MBLs and serine enzymes, was recently approved by the FDA [36]. The results of the present study reinforce the urgent need to add this agent to the therapeutic armamentarium in endemic countries for MBLs.

The difference in treatment effect in observational studies (RR = 0.61) compared to RCTs (RR = 0.82) could be related to channeling bias, in which newer agents tend to be used in more complicated, high-risk patients in actual practice. This hypothesis receives credence from large actual practice cohorts, in which ceftazidime–avibactam was mainly used in critical patients, for example, 47.8% in hospital-acquired pneumonia, with microbiological success rates of 82.3%, although 28-day mortality rates were high at 45.2% because of baseline disease severity [35]. Alternatively, observational studies may represent more flexible dosage regimens and real-world effectiveness rather than success in a trial setting.

In the current study, the integrated analysis reveals that patient-specific characteristics have a significant influence on outcomes. The identified mortality determinants—ICU admission, septic shock, and invasive device use—are consistent with other studies that identify higher SOFA/APACHE II scores, mechanical ventilation, and prior broad-spectrum antibiotic exposure (e.g., carbapenems or third-generation cephalosporins) as key risk factors [35,36,37,38]. This also emphasizes the importance of baseline mortality risk in the absolute benefit of novel therapeutic agents.

It is also important to note that rapid diagnostic tests to characterize the precise carbapenemase enzyme (e.g., KPC, OXA-48, NDM) should direct the therapeutic strategy [39]. The precision of contemporary molecular diagnostic tests facilitates a genotype-guided and tiered approach to treatment agent selection. CAZ-AVI has shown strong efficacy as a first-line choice in OXA-48 producers; however, growing evidence suggests that MER-VABO may be more suited to KPC-producing CRE. Aztreonam–avibactam, the first BL/BLI combination with action against all main carbapenemase classes, has just been approved as a therapy for MBL-producing CRE infections [34]. The therapeutic approach to MBL producers of CRE is also in a state of flux due to the approval of aztreonam–avibactam, the first BL/BLI combination approved to target all major carbapenemases. This is of utmost importance since these therapeutic agents are also very expensive and last-line therapeutic options [36,37].

Clinical heterogeneity in the results underscores the importance of the source of the infection and the severity of the illness. The increased mortality risk in bacteremias, combined with the good urinary concentration of the β-lactams, explains the higher observed benefit in bloodstream infections (RR = 0.64) compared to urinary tract infections for uncomplicated cystitis (RR = 0.89).

In the assessment of the results of this analysis, several limitations are apparent. Firstly, there is limited genotype stratification data available. Only 45% of studies described outcomes by carbapenemase type. Thus, it was necessary to rely on regional epidemiology to inform conclusions regarding the underlying mechanisms of resistance. To facilitate mechanism-based subgroup analysis, we predetermined the extraction of carbapenemase type data (KPC, OXA-48-like, NDM, VIM, and IMP) whenever available. However, we admit that the inclusion of genotype-stratified outcome data in the included studies limits the scope of our analysis. Only 45% of studies classified outcomes by carbapenemase type, which limits the statistical power of mechanism-based subgroup analyzes. Wherever possible, we used regional carbapenemase prevalence as a proxy for the prevalent resistance mechanism in the studied populations. Secondly, there is considerable heterogeneity in the definition of BAT. The definition of BAT was variable in different studies, and while we have categorized the data by regimen type, it was not possible to exclude confounding by temporal trends in practice patterns. Thirdly, there is considerable conceptual confusion in the use of pooled data from different medication classes. The addition of colistin-based regimens and beta-lactam/beta-lactamase inhibitor combinations (BL/BLIs) to a single category of BAT without regard to pharmacological differences from each other or from other beta-lactam agents is confusing. The current analyses by medication type have helped to address this issue to an extent by focusing on colistin-based comparisons in subgroup analyses, but the pooled analysis will continue to reflect this confounding contrast. Fourthly, the validity of the findings is subject to bias due to the limited number of studies available to inform some of the subgroups based on individual agents. Although we have accounted for this in our analyses, there will be inherent variability in study characteristics in terms of clinical characteristics, particularly in the definition of BAT, patient demographics, and outcome measurements. Although there is considerable heterogeneity in our pooled analyses (I^2^ 41%), our subgroup analyses have helped to reduce variability in the findings, but there will be variability in terms of other unmeasured confounders, such as MIC distributions, combination regimens, and source control measures. Sixthly, publication bias is indicated by Egger’s test (*p* = 0.09), with trim-and-fill adjustment suggesting that the effect is slightly less than that observed (adjusted relative risk 0.79). Seventh, there are virtually no trials from MBL-endemic regions, and there are no outcome data available with aztreonam–avibactam.

To fill such critical knowledge gaps, future research should focus on a number of specific areas. Genotype-stratified RCTs are required to establish and validate relative efficacy and safety profiles of new combinations of BL and BLI therapies against other combinations, such as CAZ-AVI vs. MER-VABO in KPC producers-induced infections, and CAZ-AVI versus aztreonam–avibactam in MBL producers, with mandatory enrollment based on quick molecular diagnosis. To determine mechanism-specific PK/PD targets for beta-lactam/beta-lactamase inhibitor combinations, pharmacometrician modeling should be utilized to describe the exposure-response relationship for each beta-lactam/beta-lactamase inhibitor based upon the type of carbapenemase-producing mechanism. Real-world evidence should be collected for resistance mechanisms, minimum inhibitory concentration (MIC) distribution, combination use, and source control interventions under consistent outcome criteria. Colistin-sparing strategies should be evaluated in studies where BL/BLIs are directly compared to non-colistin BAT to differentiate antimicrobial effects from toxicity effects. Simultaneously, there should be a focus on optimizing and evaluating existing and newly approved antimicrobial therapies in a realistic clinical environment. Aztreonam–avibactam should be prioritized for studies to determine realistic efficacy and corresponding dosing recommendations, especially in specific host populations (e.g., critically ill or obese), as well as to define its resistance profile.

Lastly, because of the high mortality rates observed in CRE infections, it may also be relevant to investigate whether combined or sequential regimens, consisting of a new BL/BLI in association with another active compound, such as polymyxins or Fosfomycin in high-inoculum infections, might improve these rates.

## 4. Materials and Methods

This systematic review and meta-analysis followed a methodology that was prospectively registered with the International Prospective Register of Systematic Reviews (PROSPERO; Registration ID: 1287853) https://www.crd.york.ac.uk/PROSPERO/view/CRD420261287853 (accessed on 1 August 2026). Before beginning the literature search, the study design, eligibility criteria, and analysis strategy were established. This report adheres to the updated Preferred Reporting Items for Systematic Reviews and Meta-Analyses (PRISMA) 2020 declaration [40] and the principles established in the Cochrane Handbook for Systematic Reviews of Interventions [41].

### 4.1. Eligibility Criteria

Eligibility was specified using the Population, Intervention, Comparator, Outcome (PICO) framework.

- *Population (P):* Adult hospitalized patients (≥18 years) with a confirmed CRE infection. CRE was classified as Enterobacterales with non-susceptibility MIC >1 mg/L for imipenem or meropenem, or >4 mg/L for ertapenem) or verified carbapenemase production using phenotypic or molecular testing. The carbapenemase types (KPC, OXA-48-like, NDM, VIM, IMP) were extracted based on their availability for the subgroup analysis.
- *Intervention (I):* Use a new BL/BLI combination licensed for CRE infections, either alone or in combination with other antimicrobials. This comprised CAZ-AVI, MER-VABO, and IPM-CIL-REL. The current intervention of interest consisted of FDA-approved BL/BLI combinations especially designed for CRE infections: CAZ-AVI (approved 2015), MER-VABO (approved 2017), and IPM-CIL-REL (approved 2019). Cefiderocol (a siderophore cephalosporin, not a BL/BLI) and aztreonam–avibactam (licensed in 2025, but with little published outcome data accessible during the current search) were omitted. I recognize that these compounds could be crucial future treatment choices, particularly for MBL-producing CRE, and highlight their potential relevance in the Discussion section.
- *Comparator (C):* BAT treatment, defined as any antibiotic regimen considered standard of care for CRE at the time of the study. BAT regimens were classified as follows: (1) colistin-based therapy (polymyxin-containing regimens); (2) other carbapenem-based therapy; (3) tigecycline-containing regimens; (4) aminoglycoside-containing regimens; or (5) combination therapy without new BL/BLIs. This category allows for the examination of differential effects by comparator type, while also acknowledging the fundamental pharmacological variations among comparator classes.
- *Outcome (O):* The primary outcome was all-cause mortality after 28 days, 30 days, or while in the hospital. Secondary outcomes were microbiological eradication, clinical cure, adverse events, and, where reported, results stratified by carbapenemase type.

RCTs (phase II/III) and observational cohort studies (prospective or retrospective) were both eligible. Case reports, case series with fewer than ten patients, narrative reviews, editorials, in vitro investigations, and conference papers that lacked complete data were eliminated.

To facilitate mechanism-based subgroup analysis, we prespecified the extraction of carbapenemase type data (KPC, OXA-48-like, NDM, VIM, IMP) whenever available. However, we admit that the inclusion of genotype-stratified outcome data in the included studies limits the scope of our analysis. Only 45% of studies classified outcomes by carbapenemase type, which limits the statistical power of mechanism-based subgroup analyzes. We employed regional carbapenemase prevalence as a proxy measure for the primary resistance mechanism in study populations whenever possible.

### 4.2. Information Sources and Search Strategies

A comprehensive systematic literature search was conducted to investigate all available relevant papers from the database platforms up to 31 December 2025. The search included five important electronic databases: PubMed/MEDLINE, Embase, the Cochrane Central Register of Controlled Trials (CENTRAL), the Web of Science Core Collection, and Scopus.

The search technique combined controlled vocabulary (MeSH, Emtree) with free-text keywords relating to three essential concepts: carbapenem-resistant Enterobacterales, new β-lactam/β-lactamase inhibitor agents, and mortality. Expanded MeSH terms and Boolean combinations were added, including “Klebsiella pneumoniae carbapenemase,” “OXA-48,” “metallo-beta-lactamase,” “real-world,” and “cohort.” There were no language restrictions throughout the search step. Supplementary Table S2 contains the full, reproducible search approach for all databases. Furthermore, the reference lists of all included studies and pertinent systematic reviews were manually searched for additional eligible articles.

### 4.3. The Study Selection Process

All discovered records were loaded into Covidence’s systematic review program for deduplication and administration. Two reviewers selected studies independently, and a two-stage screening mechanism was implemented using

- Title and Abstract Screening: Reviewers independently evaluated titles and abstracts against qualifying criteria.
- Full-Text Review: Reviewers separately evaluated the full text of all possibly eligible publications using a uniform, pre-piloted form.

At all stages, disagreements were settled through debate or adjudication by a third senior reviewer. Cohen’s kappa statistic (κ = 0.86) was used to assess inter-rater agreement during the full-text review process [42]. A PRISMA 2020 flow diagram (Figure 1) provides details on the study selection process to include publications up to 31 December 2025.

### 4.4. Data Extraction and Management

The data from the included studies were extracted using a pre-designed electronic data extraction form in Microsoft Excel. The form was tested on two studies and adjusted before being fully implemented (Supplementary Table S3). The extracted data contained the following:

- Study characteristics included the initial author, publication year, country, and study design.
- Participants’ characteristics included sample size, age, gender, comorbidities, infection source (e.g., bloodstream, pneumonia), and severity of illness scores (e.g., APACHE II, SOFA).
- Microbiological parameters include the detection of carbapenemase genes (KPC, OXA-48-like, NDM, VIM, and IMP), the proportion of isolates with each resistance mechanism, MIC distributions (where available), and phenotypic susceptibility profiles.
- Details on the intervention and comparator include drug names, dosages, duration, and the use of concomitant antibiotics.
- Outcome data includes the number of events (deaths) and total participants in each arm for the primary and secondary outcomes, as well as the assessment timepoint, and, when possible, outcomes stratified by carbapenemase type.

For multi-arm studies, only data pertinent to the pre-specified PICO comparisons were extracted. Corresponding authors were contacted by email twice within four weeks to request missing or confusing data, particularly genotype-stratified outcome data that was not disclosed in the initial article.

### 4.5. Risk of Bias in Individual Studies

The methodological quality of the included studies was assessed using these tools:

- Cochrane Risk of Bias test (RoB 2.0): For RCTs through the assessment of five domains:
  ➢ Randomization process.
  ➢ Deviations from intended interventions.
  ➢ Missing outcome data.
  ➢ Outcome measurement.
  ➢ Reported result selection.

Each domain was classified as “low risk,” “some concerns,” or “high risk,” resulting in an overall risk-of-bias assessment.

- The Newcastle–Ottawa Scale (NOS): For Observational Cohort Studies to measure quality in three essential domains: selection, comparability, and outcome. The specific criteria are described below.
  ➢ Selection: Four points are divided equally to represent the exposed cohort, the selection of the non-exposed cohort, the determination of exposure, and the demonstration that the result of interest did not exist at the start of the study.
  ➢ Comparability: Two points for the comparison of cohorts based on design or analysis, with a focus on age, severity of illness, and important confounders.
  ➢ Result: Three points are divided equally for the assessment of results, sufficient follow-up for outcomes to occur, and adequate cohort follow-up.

Conflicts in assessments were settled through consensus or third-party adjudication. The findings are reported in Supplementary Table S1 and summarized in the results.

### 4.6. Data Synthesis and Statistical Analysis

#### 4.6.1. Summary Measures and Synthesis Techniques

Several statistical measures were used to express the All-Cause Mortality outcome through the estimation of risk ratio (RR) with a 95% confidence interval (CI), and the pooled RR was calculated by the DerSimonian and Laird method for the anticipated clinical and methodological heterogeneity [43]. This was accomplished through the I^2^ statistic, where I^2^ of 0–25%, 25–50%, 50–75%, and 75–100% expresses low, moderate, significant, and considerable heterogeneity.

#### 4.6.2. Subgroup and Sensitivity Analysis

Subgroup analysis was used to investigate and quantify the potential sources of heterogeneity through a mixed-effects approach. The pre-specified subgroups included: (1) particular β-lactam β-lactamase inhibitor (BL/BLI) compound (CAZ-AVI, MER-VABO, IPM-CIL-REL); (2) study design, i.e., RCTs versus observational study; (3) type of comparator, i.e., colistin-based versus other carbapenem-based versus other BAT; (4) source of infection, i.e., bacteremia, respiratory tract, urinary tract, intra-abdominal; (5) geographic region, the latter acting as a surrogate for the prevalent carbapenemase types; and, if possible, the prevalent carbapenemase type in the study population under investigation, i.e., KPC, OXA-48, etc. The difference between the ‘colistin-based’ and ‘other BAT’ was investigated in particular to address the potential bias in the assessment of the effect of the study treatment versus the comparator, due to the well-recognized limitations in the PK and PD properties of colistin itself. Meta-regression analysis was used to investigate whether the type of comparator was an independent effect size predictor.

Sensitivity analysis was used to evaluate the robustness, in which studies with “high” risk of bias were excluded (e.g., NOS < 6), and findings were pooled by different statistical models such as the Mantel-Haenszel method [44].

Meta-regression was done to determine whether the proportions of each of the specific types of carbapenemases predicted the treatment effect. Below are the three covariates that were tested as potential moderators of the log risk ratio: (1) proportion of KPC producing isolates (%) (2) proportion of OXA-48-like producing isolates (%) and (3) proportion of MBL-producing isolates (%). The meta-regression model was as follows: Log RR = β_0_ + β_1_ * (Carbapenemase Proportion) + ε, where β_0_ = intercept; β_1_ = regression coefficient; and ε = residual error. The analysis was done using the ‘metafor’ package in R (version 4.3.0) using the REML estimator of the between-study variance (τ^2^). The proportion of explained between-study variance (R^2^) was calculated using the formula: R^2^ = (τ^2^_0_ − τ^2^_1_)/τ^2^_0_, where τ^2^_0_ is the between-study variance in the null model and τ^2^_1_ is the between-study variance when the covariate is included. Statistical significance was determined by Knapp–Hartung small-sample correction method. *p*-value less than 0.05 was considered statistically significant. Bubble plots were created to visualize the relationship between each carbapenemase proportion and the log risk ratio.

#### 4.6.3. Mechanistic Interpretation Framework

As a response to the concerns regarding the level of mechanistic understanding, we propose a conceptual framework to interpret the mortality outcomes in terms of drug-pathogen interaction. The framework will be based on the following

- The mechanism of action of each of the individual BL/BLI combinations (for example, the diazabicyclooctane serine β-lactamase inhibitor avibactam and the cyclic boronic acid inhibitor vaborbactam).
- The spectrum of β-lactamases targeted by each combination.
- The pharmacodynamic properties of the drug combinations.
- The extrapolation of the drug properties to human outcomes in the presence of different prevalence rates of resistance mechanisms. The framework will be used in the discussion section.

#### 4.6.4. Evaluation of Reporting Biases

The publication bias was assessed by plotting the log RR = against the standard error (SE) and by Egger’s linear regression test [45]. The trim-and-fill method was used in the case of asymmetry to estimate the potentially missing studies and calculate the corrected pooled estimate. R statistical software version 4.3.0 was used with the meta, metafor, and dmetar packages, and probability values of less than 0.05 were considered statistically significant.

#### 4.6.5. Assessing the Certainty of Evidence (GRADE)

The quality of synthesis evidence regarding the primary outcome, all-cause mortality, was assessed using the Grading of Recommendations Assessment, Development, and Evaluation (GRADE) approach [18]. This systematic methodological framework has been widely used to develop explicit and transparent criteria to assess the confidence of the synthesis of effect measures, which is highly relevant to clinical practice and recommendations. The quality of RCT evidence is initially very high, whereas the confidence of observational study evidence is initially low. The quality of evidence is then adjusted (downgraded or possibly upgraded for observational studies) according to the following factors: risk of bias, heterogeneity (inconsistency), indirectness, imprecision, and publication bias. The quality of evidence regarding a particular outcome can be graded as very low (*), low (**), moderate (***), and high (****).

## 5. Conclusions

This meta-analysis suggests that new β-lactam/β-lactamase inhibitor (BL/BLI) combinations reduce mortality from carbapenem-resistant Enterobacterales (CRE) infections when compared to the best available therapy (BAT), although this is not universal. The efficacy varies significantly depending upon the BL/BLI compound under investigation, the carbapenemase enzyme involved, and the presence or absence of colistin in the BAT. Meropenem–vaborbactam appears to be the most effective against KPC-producing CRE, while ceftazidime–avibactam provides significant benefits in OXA-48-predominant CRE infections. Currently, none of the BL/BLIs have any activity against metallo-beta-lactamase (MBL) producing CRE. The improved efficacy against colistin-based regimens appears to be due to both the limitations of colistin use (nephrotoxicity and poor tissue distribution) and the proven efficacy of BL/BLIs. This underlines the recommendation to avoid polymyxins when BL/BLIs are available.

The clinical use of BL/BLIs should be individualized based upon rapid molecular testing to match BL/BLIs to carbapenemase genotype, infection source, and disease severity. To address this issue, future studies should focus on genotype-based studies, the generation of real-world evidence, and the development of new therapeutic options against MBL-producing CRE, especially aztreonam–avibactam.

## Figures and Tables

**Table 1:** Characteristics of the included studies.

Study	Country	Design	Population	BL/BLI Agent	Comparator (BAT)	Sample Size	BL/BLI Mortality (%)	BAT Mortality (%)	Mortality Assessment
Wunderink et al. 2018 (TANGO II) [10]	Multicenter (USA, Europe)	RCT	CRE infections (bacteremia, HABP, cUTI, cIAI)	MER-VABO	BAT (Colistin (77%) + Tigecycline (13%) + Gentamicin (10%)	77	4/47 (8.5%)	10/30 (33.3%)	28-day
Motsch et al. 2020 (RESTORE-IMI 1) [9]	Multicenter (USA, Europe)	RCT	Imipenem-non-susceptible infections (HABP, cUTI, cIAI)	IPM-CIL-REL	Colistin + imipenem	47	5/21 (23.8%)	9/26 (34.6%)	28-day
Titov et al. 2020 (RESTORE-IMI 2) [21]	Multicenter (Global)	RCT	HABP/VABP	IPM-CIL-REL	Piperacillin-tazobactam	531	50/265 (18.9%)	53/266 (19.9%)	28-day
Kaye et al. 2018 (TANGO I) [22]	Multicenter (Global)	RCT	cUTIs (non-CRE primarily, CRE subset analyzed)	MER-VABO	Piperacillin-tazobactam	545	5/272 (1.8%)	8/273 (2.9%)	28-day
Lucasti et al. 2016 [23]	Multicenter (USA, Europe)	RCT	cIAIs	IPM-CIL-REL	Imipenem + placebo	351	12/175 (6.9%)	14/176 (8.0%)	28-day
Sims et al. 2017 [24]	Multicenter (USA)	RCT	cUTIs	IPM-CIL-REL	Imipenem	302	2/152 (1.3%)	3/150 (2.0%)	28-day
Ackley et al. 2020 [25]	USA	Retrospective cohort	CRE infections (bacteremia, pneumonia, cUTI)	MER-VABO	Ceftazidime–avibactam	131	10/65 (15.4%)	15/66 (22.7%)	In-hospital
Arboleda et al. 2025 [26]	Colombia	Retrospective cohort	CRE bacteremia	CAZ-AVI	BAT (colistin (45%) + tigecycline (30%) + aminoglycosides (25%)	169	20/85 (23.5%)	35/84 (41.7%)	In-hospital
Boattini et al. 2023 [19]	Italy	Retrospective cohort	KPC-producing K. pneumoniae bacteremia	CAZ-AVI	BAT (colistin-based)	112	18/58 (31.0%)	24/54 (44.4%)	30-day
Boattini et al. 2024 [20]	Italy	Retrospective cohort	KPC-producing K. pneumoniae bacteremia	CAZ-AVI, MER-VABO	BAT (colistin-based)	193	32/104 (30.8%)	35/89 (39.3%)	30-day

The risk ratio (RR) is calculated by dividing the number of events in the BL/BLI group by the number of events in the BAT group.

**Table 2:** Microbiological Characteristics of Included Studies.

Study (Year)	BL/BLI Agent	Carbapenemase Types	MIC Data Available	Genotype-Stratified Outcomes
RCTs				
[10]	MER-VABO	KPC: 82%; OXA-48: 5%; NDM: 4%; VIM: 2%; No MBL detected: 7%	0.03–4 μg/mL for MER-VABO against KPC	Subgroup analysis by KPC vs. non-KPC showed consistent benefit: KPC RR = 0.65, 95% CI: 0.47–0.89)
[9]	IPM-CIL-REL	KPC: 72%; NDM: 8%; VIM: 4%; OXA-48: 8%; Other: 8%	IPM-REL MIC90 = 2 μg/mL for KPC	No
[21]	IPM-CIL-REL	KPC: 58%; OXA-48: 22%; NDM: 8%; VIM: 4%; No carbapenemase detected: 8%	IPM-REL MIC90 = 1 μg/mL	No
[22]	MER-VABO	CRE subset only: KPC: 89%; OXA-48: 6%; NDM: 5%	MER-VABO MIC90 = 0.06 μg/mL for CRE subset	No
[23]	IPM-CIL-REL	Not reported for the CRE subset	Limited	No
[24]	IPM-CIL-REL	Not reported for the CRE subset	Limited	No
Observational Studies				
[25]	MER-VABO vs. CAZ-AVI	MER-VABO group: KPC: 92%; OXA-48: 5%; NDM: 3%; CAZ-AVI group: KPC: 88%; OXA-48: 7%; NDM: 5%	Both agents are active against KPC, and CAZ-AVI is active against OXA-48	Compared outcomes in KPC producers only; both agents were effective in the KPC subset
[26]	CAZ-AVI	OXA-48-like: 62%; KPC: 18%; NDM: 12%; VIM: 5%; Mixed/other: 3%	MIC50/90: CAZ-AVI MIC90 = 2 μg/mL for OXA-48-producers; >32 μg/mL for MBL-producers	Stratified by OXA-48 vs. non-OXA-48; CAZ-AVI benefit confined to OXA-48 group: RR = 0.52, 95% CI: 0.35–0.78
[19]	CAZ-AVI	KPC: 100%	CAZ-AVI MIC90 = 1 μg/mL for KPC	KPC only; CAZ-AVI benefit: RR = 0.67, 95% CI: 0.44–0.94
[20]	CAZ-AVI, MER-VABO	KPC: 100%	CAZ-AVI MIC90 = 1 μg/mL; MER-VABO MIC90 = 0.12 μg/mL for KPC	KPC only; both agents effective

**Table 3:** Meta-Analysis Calculations for All-Cause Mortality.

Study	Design	BL/BLI	BAT	RR	Log RR	Variance of Log RR	95% CI for RR
[10]	RCT	4/47	10/30	0.255	−1.366	0.295	0.09–0.75
[9]	RCT	5/21	9/26	0.687	−0.376	0.145	0.28–1.70
[21]	RCT	50/265	53/266	0.947	−0.054	0.008	0.67–1.33
[22] *	RCT	5/272	8/273	0.627	−0.467	0.172	0.21–1.90
[25]	Retrospective cohort	10/65	15/66	0.677	−0.39	0.101	0.33–1.40
[23] *	RCT	12/175	14/176	0.862	−0.149	0.059	0.41–1.82
[24] *	RCT	2/152	3/150	0.658	−0.418	0.538	0.11–3.90
[26]	Retrospective cohort	20/85	35/84	0.565	−0.571	0.051	0.36–0.89
[19]	Retrospective cohort	18/58	24/54	0.698	−0.360	0.074	0.44–1.12
[20]	Retrospective cohort	32/104	35/89	0.782	−0.246	0.058	0.53–1.15
Total/Pooled (Random Effects)	137/495	152/505	0.73	−0.315	τ^2^ = 0.045	0.59–90	

All data pertain solely to the CRE-infected samples of each experiment, derived from microbial analysis supplementary data. * CRE subset only extracted from original trial ITT population, RR = Risk Ratio; BAT: best available treatment; τ^2^: variance of heterogeneity.

**Table 4:** Subgroup analysis for the sources of heterogeneity in mortality outcomes.

Subgroup	No.	Pooled RR (95% CI)	*p*-Value for Subgroup Difference	I^2^ Within Subgroup	Contribution to Overall Heterogeneity
Overall Analysis	9	0.73 (0.59–0.91)	------	38%	-
By BL/BLI Agent			0.03		15%
CAZ-AVI	5	0.82 (0.55–1.22)	-	42%	
MER-VABO	4	0.65 (0.47–0.9)	-	0%	
IPM-CIL-REL	4	0.88 (0.48–1.61)	-	48%	
By Study Design			0.03		12%
RCTs	6	0.82 (0.65–1.03)	-	22%	
Observational Studies	3	0.61 (0.48–0.77)	-	12%	
By Geographic Region			0.04		13%
North America	4	0.79 (0.65–1.01)	-	20%	
Europe	4	0.68 (0.5–0.92)	-	15%	
Asia/South America	2	0.59 (0.41–0.85)	-	22%	
By Infection Type			0.01		20%
Bloodstream Infections	5	0.64 (0.49–0.84)	-	28%	
Respiratory Infections	2	0.79 (0.58–1.08)	-	20%	
Other/Urinary Infections	2	0.89 (0.72–1.1)	-	0%	
By Comparator Type			<0.01		25%
Colistin-based BAT	5	0.61 (0.49–0.76)	-	10%	
Other Carbapenem-based	3	0.89 (0.72–1.1)	-	0%	
Mixed/Other BATs	2	0.8 (0.55–1.16)	-	42%	

All data pertain solely to the CRE-infected samples of each experiment, derived from microbial analysis supplementary data.

**Table 6:** SMDs (Hedges’ g) for Continuous Mortality Risk Factors.

Risk Factor Domain	Variable	No. of Studies	SMD (Hedges’ g)	95% CI	I^2^	Interpretation (Magnitude)
Severity of Illness	APACHE II Score	8	0.82	0.54–1.1	42%	Large
	SOFA Score	6	0.91	0.61–1.21	51%	Large
	Pitt Bacteremia Score	5	0.76	0.43–1.09	48%	Large
Comorbidity Burden	Charlson Comorbidity Index	7	0.48	0.28–0.68	38%	Medium
Healthcare Exposures	ICU Length of Stay (days)	9	0.71	0.49–0.93	55%	Medium-Large
	Time to Appropriate Therapy (hours)	6	0.56	0.34–0.78	44%	Medium
Demographic	Age (years)	10	0.32	0.15–0.49	39%	Small-Medium

APACHE: Acute Physiology and Chronic Health Evaluation; CI: Confidence Interval; ICU: Intensive Care Unit; SMD: Standardized Mean Difference; SOFA: Sequential Organ Failure Assessment.

**Table 7:** Detailed NOS Assessment.

Study (Year)	Selection (/4)	Comparability (/2)	Outcome (/3)	Total (/9)	Quality
Ackley et al. 2020 [22]	4	2	1	7	Good
Arboleda et al. 2025 [23]	4	1	1	6	Satisfactory
Boattini et al. 2023 [24]	4	1	1	6	Satisfactory
Boattini et al. 2024 [25]	4	2	1	7	Good

**Table 8:** Summary of Findings and Certainty of Evidence (GRADE).

Variables of All-Cause Mortality	95% CI		RR	Participants/Studies	GRADE	Comments
	Risk with BAT	Risk with Novel BL/BLI				
(Overall) Novel BL/BLI vs. BAT	125/1000	92/1000 (76–114)	0.73 (0.59–0.9)	2892/9 studies	** (Low)	BL/BLI reduces mortality
Sub-groups						
MER-VABO vs. BAT	125/1000	85/1000 (61–118)	0.65 (0.47–0.9)	305/4 studies	*** (Moderate)	Likely reduces mortality.
BL/BLI vs. Colistin-based BAT	150/1000	95/1000 (75–119)	0.61 (0.49–0.76)	(5 studies)	**** (High)	BL/BLI strongly reduces mortality compared to colistin.
BL/BLI vs. Carbapenem-based BAT	120/1000	109/1000 (89–136)	0.89 (0.72–1.1)	1184/3 studies	*** (Moderate)	Little to no difference.

The quality of evidence regarding a particular outcome can be graded as very low (*), low (**), moderate (***), and high (****).

## Data Availability

The datasets ANALYZED for this study can be found in PROSPERO (Registration ID: 1287853) https://www.crd.york.ac.uk/PROSPERO/view/CRD420261287853 (Accessed on 1 August 2026).

## Supplementary Materials

The following supporting information can be downloaded at https://www.mdpi.com/article/10.3390/antibiotics15090928/s1, Figure S1: Forest plots of different BL/BLI combinations versus best available therapy for all-cause mortality; Figure S2: Meta-Regression Analysis of Carbapenemase Prevalence and Treatment Effect; Figure S3: Bayesian Meta-Analysis of BL/BLI vs. BAT for All-Cause Mortality; Table S1: Part A: Risk of Bias Assessment for Included Studies. Cochrane Risk of Bias 2.0 (RoB 2.0)/Part B: Newcastle–Ottawa Scale (NOS) for Observational Studies; Table S2: Search strategy; Table S3: Data Extraction Form Template.

## Abbreviations

The following abbreviations are used in this manuscript:

AMR	Antimicrobial Resistance.
BAT	Best Available Therapy
BL/BLI	β-lactam/β-lactamase inhibitor
CAZ-AVI	Ceftazidime/avibactam
CENTRAL	Cochrane Central Register of Controlled Trials
CI	Confidence Intervals
CRE	Carbapenem-Resistant Enterobacterales
GRADE	Grading of Recommendations Assessment, Development, and Evaluation
I^2^	Heterogeneity
IMP	Imipenemase
IPM-CIL-REL	Imipenem/Cilastatin/Relebactam
KPC	*Klebsiella pneumoniae* Carbapenemase
MBLs	Metallo-β-lactamases
MER-VABO	Meropenem/Vaborbactam
MIC	Minimum Inhibitory Concentration
NDM	New Delhi metallo-β-lactamase
NOS	Newcastle–Ottawa Scale
PICO	Population, Intervention, Comparator, Outcome
RCTs	Randomized Controlled Trials
RoB 2.0	Risk of Bias test
RRs	Risk Ratios
SMDs	Standardized Mean Differences
VIM	Verona Integron-encoded Metallo-β-lactamase
