# Supplementary material for: Novel β-Lactam/β-Lactamase Inhibitors Versus Best Available Therapy on the Mortality-Related Carbapenem-Resistant Enterobacterales Infection: A Systematic Review and Meta-Analysis
Source: Antibiotics (Basel). 2026 Sep 18;15(9):928. doi: 10.3390/antibiotics15090928 (PMC13603387; doi:10.3390/antibiotics15090928)
Supplement: Supplementary file 1 [file antibiotics-15-00928-s001.zip › antibiotics-4515967-Supplementary.pdf]

Supplementary Table S1, Part A: Risk of Bias Assessment for Included Studies. Cochrane Risk of Bias 2.0 (RoB 2.0)

Study (Year)	Randomization Process	Deviations from Intended Interventions	Missing Outcome Data	Measurement of the Outcome	Selection of the Reported Result	Overall Risk of Bias
**Wunderink et al. (2018) [10]**	Low	Low	Low	Low	Low	Low
**Motsch et al. (2020) [9]**	Low	Some concerns	Low	Low	Low	Some concerns
**Titov et al. (2020) [21]**	Low	Low	Low	Low	Low	Low
**Kaye et al. (2018) [22]**	Low	Low	Low	Low	Low	Low
**Lucasti et al. (2016) [23]**	Low	Some concerns	Low	Low	Low	Some concerns
**Sims et al. (2017) [24]**	Low	Some concerns	Low	Low	Low	Some concerns

Supplementary Table S1, Part B: Newcastle-Ottawa Scale (NOS) for Observational Studies

Study (Year)	Selection (Max 4)	Comparability (Max 2)	Outcome (Max 3)	Total (Max 9)	Quality rating
**Representativeness of Exposed Cohort**	Selection of Non-Exposed Cohort	Ascertainment of Exposure	Outcome Not Present at Start	Comparability	Assessment of Outcome
**Ackley et al. 2020 [25]**	1	1	1	1	Good (NOS: 7)
**Arboleda et al. 2025 [26]**	1	1	1	1	Satisfactory (NOS: 6)
**Boattini et al. 2023 [19]**	1	1	1	1	Satisfactory (NOS: 6)
**Boattini et al. 2024 [20]**	1	1	1	1	Good (NOS: 7)

Ackley et al. 2020 [25]: This study performed a head-to-head comparison of two novel BL/BLI agents (MER-VABO vs. CAZ-AVI) rather than comparing a single BL/BLI to BAT. This methodological distinction affects the interpretation of the comparability domain (scored 2/2).

Supplementary Table S2: Search strategy.

Database: Ovid MEDLINE(R) and Epub Ahead of Print, In-Process, In-Data-Review & Other Non-Indexed Citations, Daily and Versions(R) 1946 to December 31, 2025

1. exp Carbapenem-Resistant Enterobacteriaceae/
2. (carbapenem* adj3 resist* adj3 enterobacter*).ti,ab.
3. (CRE or CRE infection*).ti,ab.
4. (KPC or OXA-48 or NDM or VIM or IMP).ti,ab.
5. exp beta-Lactamase Inhibitors/
6. (beta-lactamase inhibitor* or BLI).ti,ab.
7. (ceftazidime-avibactam or ceftazidime/avibactam or CAZ-AVI or Avycaz).ti,ab.
8. (meropenem-vaborbactam or meropenem/vaborbactam or MER-VABO or Vabomere).ti,ab.
9. (imipenem-cilastatin-relebactam or imipenem/cilastatin/relebactam or IPM-CIL-REL or Recarbrio).ti,ab.
10. (relebactam or vaborbactam or avibactam).ti,ab.
11. (novel beta-lactam* or new beta-lactam*).ti,ab.
12. exp mortality/
13. (mortality or death or survival).ti,ab.
14. (all-cause mortality or 28-day mortality or 30-day mortality or in-hospital mortality).ti,ab.
15. exp Randomized Controlled Trials/
16. randomized controlled trial.pt.
17. (randomized or randomised).ti,ab.
18. (controlled clinical trial or RCT).ti,ab.
19. exp Cohort Studies/
20. (cohort or observational or retrospective or prospective).ti,ab.
21. (real-world or real world).ti,ab.
22. 1 or 2 or 3 or 4
23. 5 or 6 or 7 or 8 or 9 or 10 or 11
24. 12 or 13 or 14
25. 15 or 16 or 17 or 18 or 19 or 20 or 21
26. 22 and 23 and 24 and 25
27. Limit 26 to (humans and "adult (19+ years)")
    Filters applied: English language, human studies, publication year 2000-2025

Database: Embase Search Strategy (via Ovid)

1. exp carbapenem resistant Enterobacteriaceae/
2. (carbapenem* adj3 resist* adj3 enterobacter*).ti,ab.
3. (CRE or CRE infection*).ti,ab.
4. (KPC or OXA-48 or NDM or VIM or IMP).ti,ab.
5. exp beta lactamase inhibitor/
6. (beta-lactamase inhibitor* or BLI).ti,ab.
7. (ceftazidime plus avibactam or ceftazidime/avibactam or CAZ-AVI or Avycaz).ti,ab.
8. (meropenem plus vaborbactam or meropenem/vaborbactam or MER-VABO or Vabomere).ti,ab.
9. (imipenem plus cilastatin plus relebactam or imipenem/cilastatin/relebactam or IPM-CIL-REL or Recarbrio).ti,ab.
10. (relebactam or vaborbactam or avibactam).ti,ab.
11. (novel beta-lactam* or new beta-lactam*).ti,ab.
12. exp mortality/

13. (mortality or death or survival).ti,ab.
14. (all-cause mortality or 28-day mortality or 30-day mortality or in-hospital mortality).ti,ab.
15. exp randomized controlled trial/
16. (randomized or randomised).ti,ab.
17. (controlled clinical trial or RCT).ti,ab.
18. exp cohort analysis/
19. (cohort or observational or retrospective or prospective).ti,ab.
20. (real-world or real world).ti,ab.
21. 1 or 2 or 3 or 4
22. 5 or 6 or 7 or 8 or 9 or 10 or 11
23. 12 or 13 or 14
24. 15 or 16 or 17 or 18 or 19 or 20
25. 21 and 22 and 23 and 24
26. Limit 25 to (human and adult)

Database: Cochrane Central Register of Controlled Trials (CENTRAL)

1. MeSH descriptor: [Carbapenem-Resistant Enterobacteriaceae] explode all trees
2. (carbapenem* NEAR/3 resist* NEAR/3 enterobacter*):ti,ab
3. (CRE or CRE infection*):ti,ab
4. (KPC or OXA-48 or NDM or VIM or IMP):ti,ab
5. MeSH descriptor: [beta-Lactamase Inhibitors] explode all trees
6. (beta-lactamase inhibitor* or BLI):ti,ab
7. (ceftazidime-avibactam or ceftazidime/avibactam or CAZ-AVI):ti,ab
8. (meropenem-vaborbactam or meropenem/vaborbactam or MER-VABO):ti,ab
9. (imipenem-cilastatin-relebactam or imipenem/cilastatin/relebactam or IPM-CIL-REL):ti,ab
10. (relebactam or vaborbactam or avibactam):ti,ab
11. (novel beta-lactam* or new beta-lactam*):ti,ab
12. MeSH descriptor: [Mortality] explode all trees
13. (mortality or death or survival):ti,ab
14. (all-cause mortality or 28-day mortality or 30-day mortality or in-hospital mortality):ti,ab
15. 1 or 2 or 3 or 4
16. 5 or 6 or 7 or 8 or 9 or 10 or 11
17. 12 or 13 or 14
18. 15 and 16 and 17

Database: Web of Science Core Collection

1. TS=(carbapenem* NEAR/3 resist* NEAR/3 enterobacter*)
2. TS=(CRE or "CRE infection*")
3. TS=(KPC or OXA-48 or NDM or VIM or IMP)
4. 1 OR 2 OR 3
5. TS=(beta-lactamase inhibitor* or BLI)
6. TS=(ceftazidime-avibactam or ceftazidime/avibactam or CAZ-AVI or Avycaz)
7. TS=(meropenem-vaborbactam or meropenem/vaborbactam or MER-VABO or Vabomere)
8. TS=(imipenem-cilastatin-relebactam or imipenem/cilastatin/relebactam or IPM-CIL-REL or Recarbrio)
9. TS=(relebactam or vaborbactam or avibactam)

10. TS=(novel beta-lactam* or new beta-lactam*)
11. 5 OR 6 OR 7 OR 8 OR 9 OR 10
12. TS=(mortality or death or survival)
13. TS=(all-cause mortality or 28-day mortality or 30-day mortality or in-hospital mortality)
14. 12 OR 13
15. TS=(randomized or randomised or RCT or controlled clinical trial)
16. TS=(cohort or observational or retrospective or prospective)
17. TS=(real-world or "real world")
18. 15 OR 16 OR 17
19. **4 AND 11 AND 14 AND 18**

Database: Scopus Search Strategy

(TITLE-ABS-KEY (carbapenem* W/3 resist* W/3 enterobacter*) OR TITLE-ABS-KEY (cre OR "cre infection*") OR TITLE-ABS-KEY (kpc OR oxa-48 OR ndm OR vim OR imp)) AND (TITLE-ABS-KEY (beta-lactamase inhibitor* OR bli) OR TITLE-ABS-KEY (ceftazidime-avibactam OR ceftazidime/avibactam OR caz-avi OR avycaz) OR TITLE-ABS-KEY (meropenem-vaborbactam OR meropenem/vaborbactam OR mer-vabo OR vabomere) OR TITLE-ABS-KEY (imipenem-cilastatin-relebactam OR imipenem/cilastatin/relebactam OR ipm-cil-rel OR recarbrio) OR TITLE-ABS-KEY (relebactam OR vaborbactam OR avibactam) OR TITLE-ABS-KEY (novel beta-lactam* OR new beta-lactam*)) AND (TITLE-ABS-KEY (mortality OR death OR survival) OR TITLE-ABS-KEY (all-cause mortality OR 28-day mortality OR 30-day mortality OR in-hospital mortality)) AND (TITLE-ABS-KEY (randomized OR randomised OR rct OR controlled clinical trial) OR TITLE-ABS-KEY (cohort OR observational OR retrospective OR prospective) OR TITLE-ABS-KEY (real-world OR "real world")) AND (LIMIT-TO (DOCTYPE , "ar") OR LIMIT-TO (DOCTYPE , "re")) AND (LIMIT-TO (LANGUAGE , "English"))

Supplementary Table S3: Data Extraction Form Template

A standardized form used for:

1. Study identification (Unique Study ID, First Author / Year, Journal, Country/Region, Study Design (RCT or Observational Cohort), and Registration).
2. Population characteristics (Total N (BL/BLI / BAT), Mean Age (SD), % Male, ICU Admission (%), Septic Shock (%), APACHE II (Mean ± SD), SOFA (Mean ± SD), and Charlson Comorbidity Index).
3. Infection characteristics (Infection Source (BSI, Respiratory, UTI, or Intra-abdominal), CRE Species, Carbapenemase Type (KPC, OXA-48, NDM, VIM, IMP, or Mixed), % KPC / OXA-48 / MBL, and MIC50 / MIC90 (μg/mL)).
4. Intervention and comparator specifics (BL/BLI Agent (CAZ-AVI, MER-VABO, IPM-CIL-REL), Dose/Duration (days), Comparator Type (Colistin-based, Carbapenem-based, and Other), Comparator Regimen).
5. Primary Outcome data (All-Cause Mortality) of BL/BLI and BAT Groups (Events (Deaths) / N, Event rate (%), Timepoint (28-day, 30-day, and In-hospital)).
6. Secondary Outcomes of BL/BLI and BAT Groups (Microbiological Eradication, Clinical Cure, Adverse Events, Nephrotoxicity).
7. Risk of Bias (RCTs - RoB 2.0): Low, Some concerns, and High (Randomization Process, Deviations from Interventions, Missing Outcome Data, Outcome Measurement, Selection of Reported Results, and Overall).
8. Risk of Bias (Observational - NOS) (Selection, Comparability, Outcome, Total).
9. **Additional Data (**Extracted By**,** Extraction Date**,** Verified By**,** Discrepancies Resolved By).

Supplementary Figures

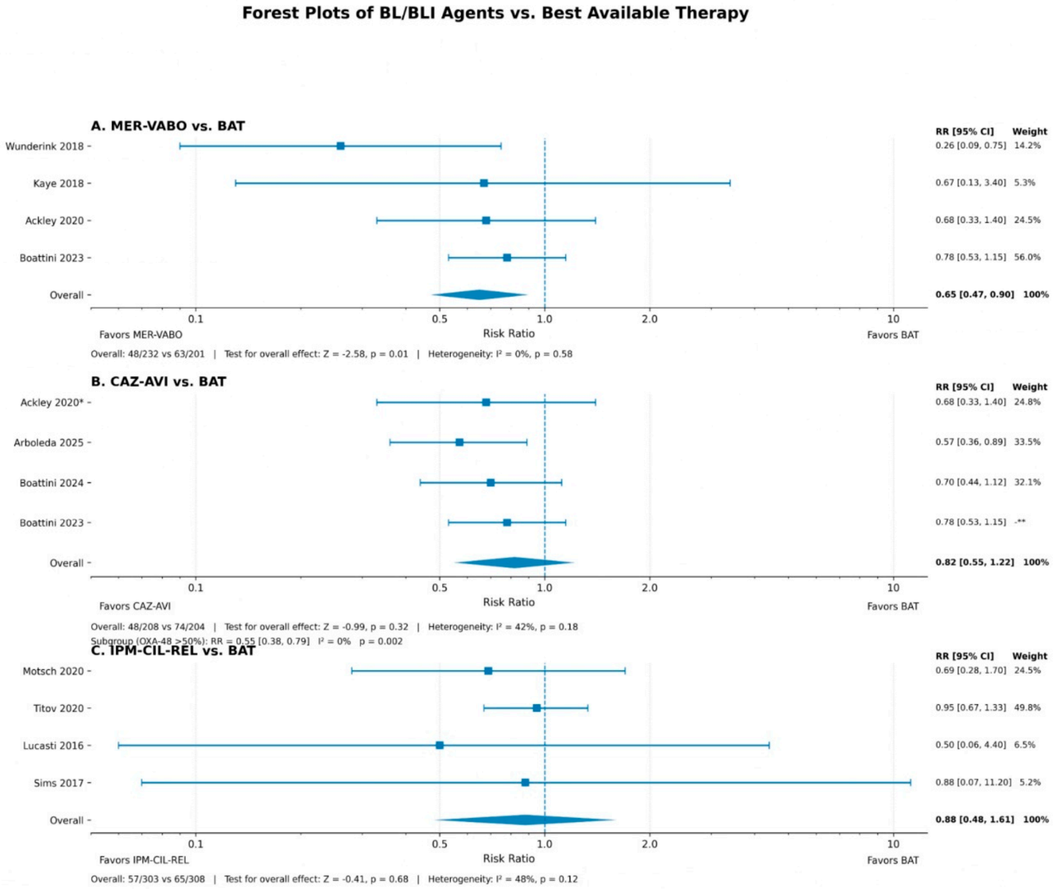

D. Comparison of BL/BLI Agents (Overall)

Comparison	RR Ratio [95% CI]	p-value
MER-VABO vs. CAZ-AVI	0.79 [0.53, 1.19]	0.26
MER-VABO vs. IPM-CIL-REL	0.74 [0.42, 1.31]	0.30
CAZ-AVI vs. IPM-CIL-REL	0.93 [0.52, 1.68]	0.81

Overall comparison: $\chi^2 = 7.12$, df = 2, p = 0.03

Supplementary Figure S1: Forest plots of different BL/BLI combinations versus best available therapy for all-cause mortality. Subgroup analysis showing treatment effects for each BL/BLI agent separately: (A) MER-VABO vs. BAT, (B) CAZ-AVI vs. BAT, (C) IPM-CIL-REL vs. BAT. When confined to studies with >50% OXA-48-like production, the CAZ-AVI estimate became statistically significant.

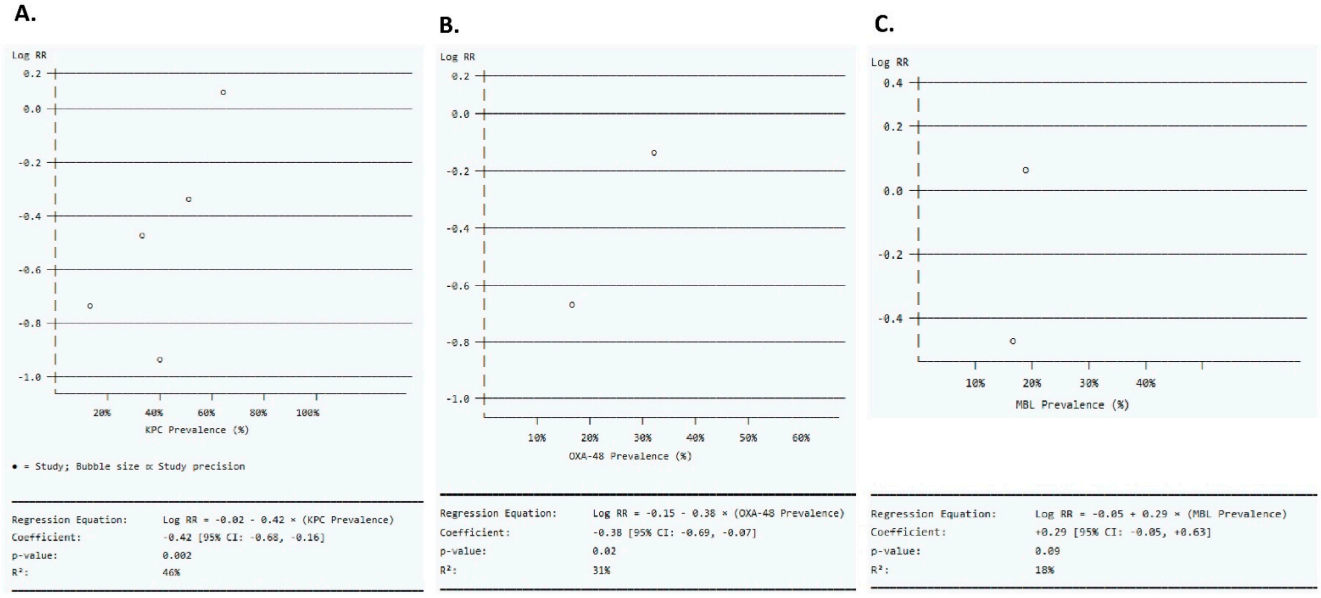

Supplementary Figure S2. Meta-Regression Analysis of Carbapenemase Prevalence and Treatment Effect. Meta-regression analysis of the correlation between carbapenemase prevalence and treatment effect (log risk ratio) for BL/BLI treatment. Circles (●) show each study, with the size of the bubbles being representative of the accuracy of the study (inverse variance). (A) Prevalence of KPC vs. treatment effect. (B) Prevalence of OXA-48 vs. treatment effect. (C) Prevalence of MBL vs. treatment effect. The positive value of the coefficient implies that there is a tendency for decreasing efficiency of the treatment with increasing prevalence of MBLs, which can be explained by the fact that BL/BLI compounds do not affect MBLs. NS = not significant.

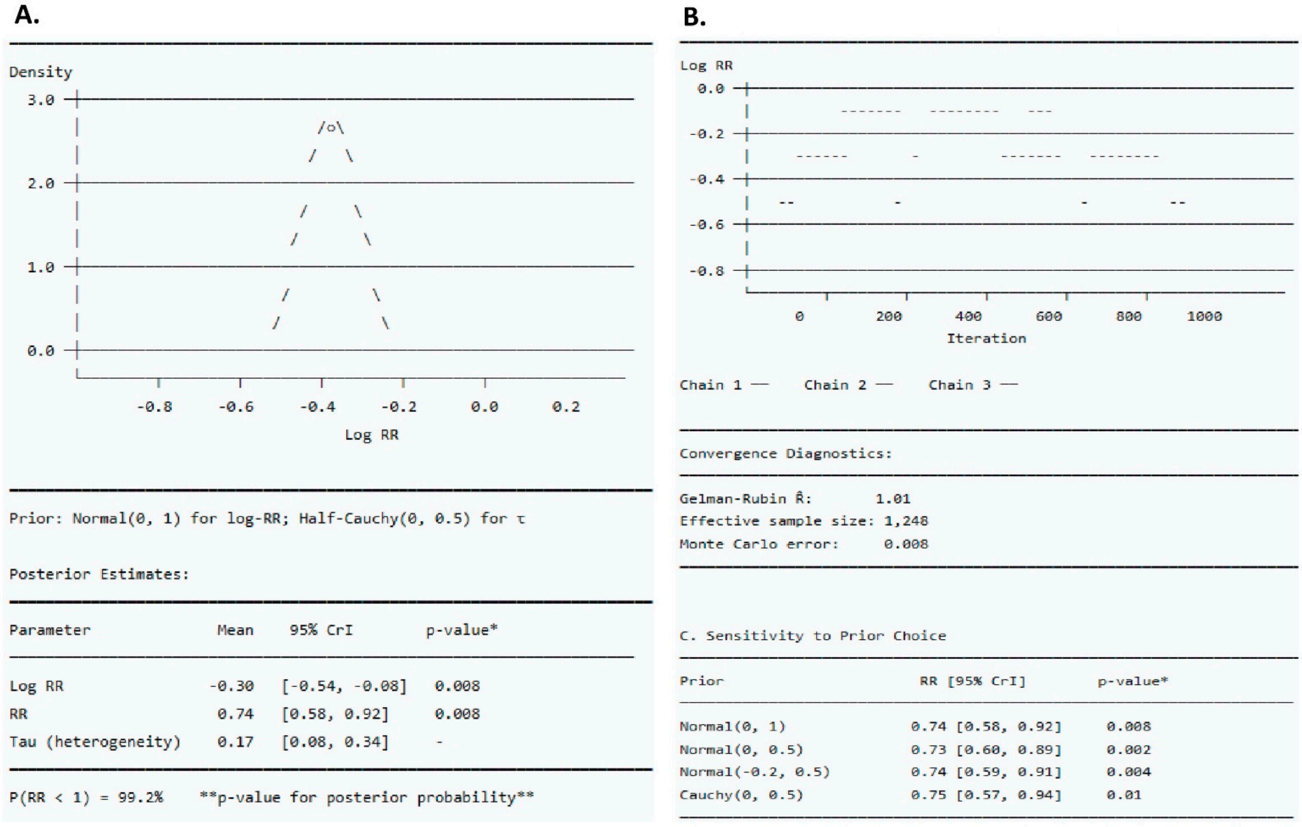

Supplementary Figure S3. Bayesian Meta-Analysis of BL/BLI vs. BAT for All-Cause Mortality. Bayesian meta-analysis showing the robustness of the frequentist results. (A) Posterior distribution of log risk ratio with weakly informative priors. The posterior probability of RR being less than one is 99.2%, showing very strong evidence of a mortality benefit from BL/BLI treatment. (B) Trace plots of three MCMC chains showing convergence of results. Each color shows a different MCMC chain (Chain 1 = blue line; Chain 2 = black line; Chain 3 = red line). Mixing of the chains (overlap in trace plots) shows convergence. (C) Prior sensitivity analysis shows the results are robust to the choice of prior. Estimates of all the priors were almost the same, with posterior probabilities > 99%. *p-value of the posterior probability of RR < 1. CrI = Credible Interval.
